# Phospholipid flipping involves a central cavity in P4 ATPases

**DOI:** 10.1038/s41598-017-17742-y

**Published:** 2017-12-15

**Authors:** M. S. Jensen, S. R. Costa, A. S. Duelli, P. A. Andersen, L. R. Poulsen, L. D. Stanchev, P. Gourdon, M. Palmgren, T. Günther Pomorski, R. L. López-Marqués

**Affiliations:** 1 Department of Plant and Environmental Sciences, University of Copenhagen, 1871 Frederiksberg, Denmark; 2 Department of Biomedical Sciences, University of Copenhagen, 2200 København, Denmark; 3 Department of Experimental Medical Science, Lund University, Lund, Sweden; 4 Department of Molecular Biochemistry, Ruhr-Universität Bochum, Bochum, Germany

## Abstract

P4 ATPase flippases translocate phospholipids across biomembranes, thus contributing to the establishment of transmembrane lipid asymmetry, a feature important for multiple cellular processes. The mechanism by which such phospholipid flipping occurs remains elusive as P4 ATPases transport a giant substrate very different from that of other P-type ATPases such as Na^+^/K^+^- and Ca^2+^-ATPases. Based on available crystal structures of cation-transporting P-type ATPases, we generated a structural model of the broad-specificity flippase ALA10. In this model, a cavity delimited by transmembrane segments TM3, TM4, and TM5 is present in the transmembrane domain at a similar position as the cation-binding region in related P-type ATPases. Docking of a phosphatidylcholine headgroup *in silico* showed that the cavity can accommodate a phospholipid headgroup, likely leaving the fatty acid tails in contact with the hydrophobic portion of the lipid bilayer. Mutagenesis data support this interpretation and suggests that two residues in TM4 (Y374 and F375) are important for coordination of the phospholipid headgroup. Our results point to a general mechanism of lipid translocation by P4 ATPases, which closely resembles that of cation-transporting pumps, through coordination of the hydrophilic portion of the substrate in a central membrane cavity.

## Introduction

P4 ATPases are ATP-fueled flippases that translocate phospholipids from the extracytosolic leaflet of biomembranes to the cytosolic leaflet, by an unknown mechanism (for a recent review see^1^). Most P4 ATPases function as a heterodimeric complex consisting of a catalytic α-subunit of 10 transmembrane (TM) segments and a supporting two-TM β-subunit of the Cell division cycle 50 (Cdc50) protein family^2^. P4 ATPases belong to the P-type ATPase superfamily of primary active transporters, which are characterized by the formation of a phosphorylated reaction cycle intermediate. P-type ATPases have a conserved structure consisting of a transmembrane domain and two large cytosolic loops that include an actuator domain (A-domain), a nucleotide-binding domain (N-domain), and a phosphorylation domain (P-domain), making it likely that their catalytic mechanism is governed by common principles. However, the phospholipid substrate of P4 ATPases is much different from the transported ligand of other well-known P-type ATPase subfamilies, which are all cation transporters, such as the sarcoplasmic reticulum Ca^2+^-ATPase (SERCA), the Na^+^/K^+^-ATPase, the plasma membrane H^+^-ATPase, and the Zn^2+^-ATPase^2,3^. With phospholipids typically being about 45 times larger than cations (e.g., phosphatidylcholine vs. unhydrated Zn^2+^), the question arises as to how such a large amphipathic molecule can be transported by the same mechanism as a metal cation. In discussions on the P4 ATPase transport mechanism, this dilemma is referred to as the ‘giant substrate problem’^4,5^.

**Figure 1:**
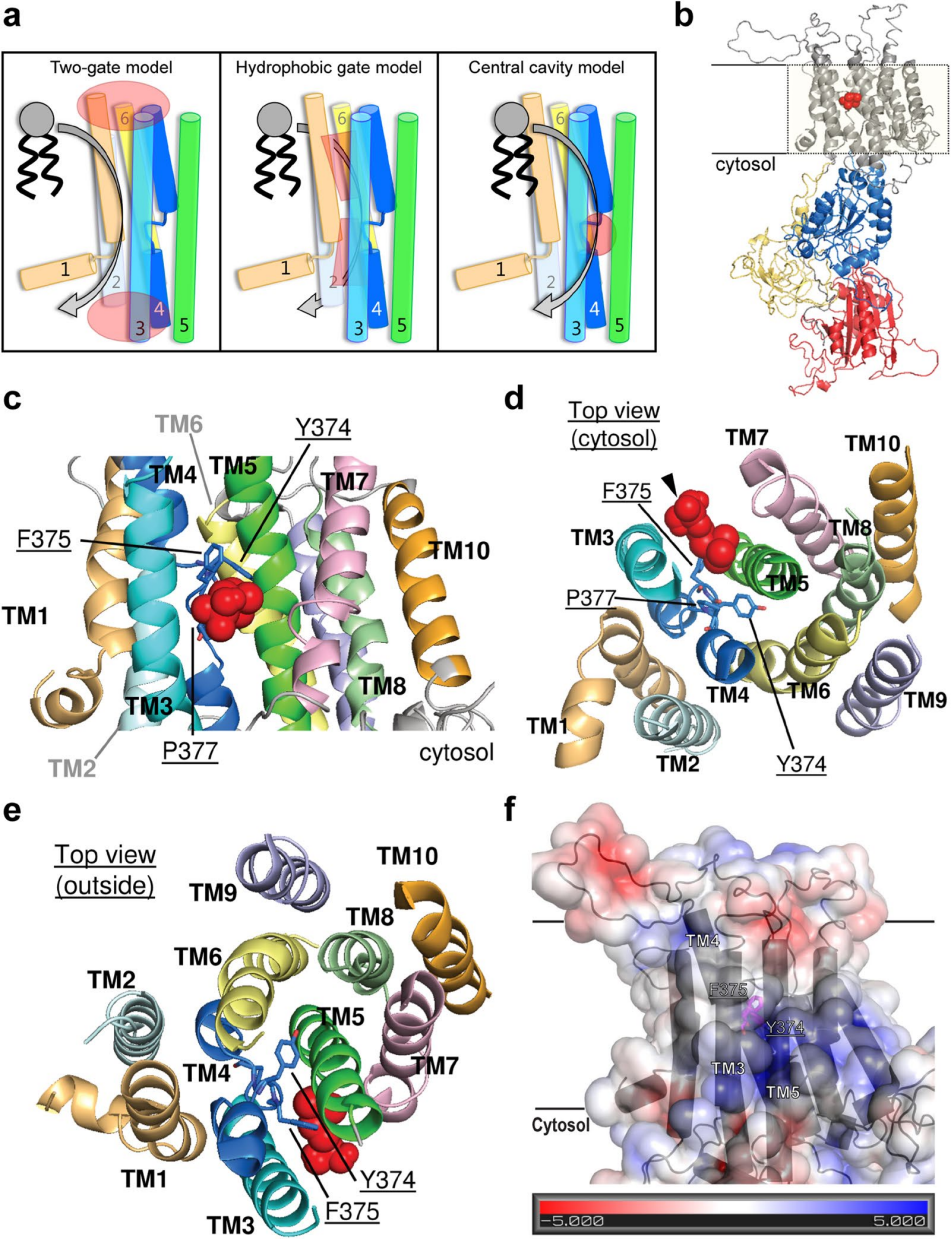
Mechanism of lipid transport by P4 ATPases. (**a**) Proposed models for the mechanism of lipid transport by P4 ATPases. The two-gate model (left) suggests that the phospholipid substrate is selected at an entry gate at the extracellular/lumenal border and at an exit gate towards the cytosol (regions highlighted in red). The hydrophobic gate model (center) proposes the presence of a hydrophobic gate inside the protein that separates alternate water-filled entry and exit cavities (highlighted in red). The central cavity model presented in this paper (right) identifies the presence of a central cavity deeply embedded in the transmembrane region (highlighted in red). For details see text. (**b**–**f**) Homology model of ALA10. An alignment of P-type ATPases and known crystal structures of Na^+^/K^+^-ATPase (pdb ID: 2ZXE), Ca^2+^-ATPase (pdb ID: 1WPG), Cu^+^-ATPase (pdb ID: 4BYG), and Zn^2+^-ATPase (pdb ID: 4UMW) in E2·P_i_ dephosphorylation transition states were used to generate a structural model of ALA10. Red spheres in (**b**–**e**) represent a phosphocholine headgroup docked inside a cavity formed between TM3, TM4 and TM5. (**b**) Full model showing the characteristic overall P-type structure with three cytoplasmic domains (A: actuator in yellow, N: nucleotide binding in red, P: phosphorylation in blue). (**c**) Lateral view of the membrane region. (**d**) View of the membrane region from the cytosol. The arrowhead marks the position of the oxygen atom in the phosphocholine group that links to the glycerol backbone in the phospholipid structure. (**e**) View of the membrane region from the extracytosolic side. (**f**) Electrostatic surface map of the membrane region with the color scale bar in the inset. Values indicate energies in kT/e. The structural model is superimposed in dark gray.

Recently, several studies have focused on identifying residues involved in determining P4 ATPase substrate specificity^6–10^. Such studies, based on mutagenesis of yeast and mammalian P4 ATPases, have resulted in two models describing the P4 ATPase lipid translocation pathway (Fig. 1a). The first model, the two-gate model, is based on studies of the P4 ATPase Dnf1p of the yeast *Saccharomyces cerevisiae* and suggests that selection of the phospholipid substrate happens in two steps^6,8,9^. The first step occurs at an entry gate formed by residues located at the extracellular/lumenal border of TM1 and TM2 and in the loop between TM3 and TM4. After this step, the phospholipid headgroup slides through a shallow groove located between TM1 and TM3 before reaching a second selective gate (exit gate) at the edges of TM1, TM2, TM3, and TM4, towards the cytosol. The second model, the hydrophobic gate model, is based on a mutagenesis study of the mammalian P4 ATPase ATP8A2, and proposes that a hydrophobic gate inside the protein separates water-filled alternating entry and exit cavities surrounded by TM1, TM2, TM4, and TM6^10^. This model is based on the observation that mutation of a conserved isoleucine residue located among other hydrophobic residues in TM4 changes the ability of the protein to release the lipid substrate and was guided by a homology model based on the crystal structure of the SERCA Ca^2+^-ATPase. In this model, the lipid headgroup is embedded in the P4 ATPase membrane region, but how the protein selects for a specific phospholipid is unresolved. Another puzzling feature of this model is that TM5 is situated behind TM4 and is clearly separated from the water-filled cavities, since previously obtained experimental evidence for ATP8A2 demonstrated that a specific conserved lysine residue in TM5 is essential for phospholipid translocation^7^. An alternative theoretical model has been recently proposed based on the plasma membrane H^+^-ATPase structure^11^. This theoretical model suggests that a water-filled cavity exists in P4 ATPases that is analogous to the one in the crystal structure of H^+^-ATPases, between TM4, TM5, and TM6. This cavity would be large enough to contain the phospholipid headgroup during transit through the membrane, while the hydrophobic hydrocarbon tail would protrude out of the protein into the core of the membrane bilayer. However, experimental data supporting the existence of such a cavity is lacking.

The P4 ATPase family in the model plant *Arabidopsis thaliana* has 12 members (ALA1-12). Among these, ALA10 strikingly functions as a broad-specificity transporter, capable of translocating multiple phospholipid substrates, such as phosphatidylserine (PS), phosphatidylcholine (PC), phosphatidylethanolamine (PE), and lyso-PC, as well as small amounts of phosphatidylglycerol (PG) and phosphatidic acid (PA)^12^. In this study, we demonstrate that, apart from these phospholipids, ALA10 can recognize and translocate fluorescently labeled sphingomyelin (SM), which contains a choline headgroup and a ceramide backbone, and is typically used as a negative control for P4 ATPase activity. To decipher the molecular basis for the unusually broad lipid recognition of ALA10, we generated a homology model based on the alignment of the protein with several previously structurally determined P-type ATPases, and tested this model using a targeted mutagenesis approach combined with lipid uptake assays in yeast. The previous two-gate and hydrophobic gate models only dealt with the entry and exit of the phospholipid headgroup. The results presented herein add a new piece to the puzzle by identifying a putative central cavity between TM3, TM4, and TM5, large enough to accommodate a phospholipid headgroup. We show that residues Y374 or F375 in ALA10 are important for recognition of the lipid substrate. Our results suggest that the central cavity might be important for controlling phospholipid specificity.

## Results

### TM4 may line an internal central cavity in ALA10

ALA10 is an unusual flippase in that it has broad specificity towards a variety of phospholipids, such as PS, PE, PC, and lyso-PC, all of which contain a glycerol backbone^12^. Given the lack of structural information of P4 ATPases to guide efforts to understand the substrate specificity of ALA10, we took advantage of the homology of this protein to P-type ATPases that have been structurally characterized. Thus, using an alignment of ALA10 with diverse P-type ATPase amino acid sequences from all classes, we modeled the structure of ALA10 based on the crystal structures of the Na^+^/K^+^-, Ca^2+^-, Cu^+^- and Zn^2+^-ATPases trapped in the transition state of dephosphorylation (E2∙P_i_) of the P-type ATPase catalytical cycle, as this is the P4 ATPase conformation expected to bind phospholipids^13^. No proton ATPase was included during modelling, due to lack of an equivalent E2∙P_i_ structure. In contrast to previous models based solely on the Ca^2+^-ATPase structure, the inclusion of several structures for P-type ATPases of different families may allow our model to account for larger structural variations, which may be shared with the flippases in certain regions. The obtained model shows an overall structural similarity to other P-type ATPases with a membrane region consisting of 10 TM helixes, and a large cytosolic domain (Fig. 1b and Supplementary Fig. 1). Also similarly to other P-type ATPases, TM4 is located in the center of the membrane region, packed between TM1, TM2, TM3, TM5, and TM6^14,15^ (Fig. 1c-e).

To identify parts of the ALA10 molecule that might be able to accommodate a phospholipid headgroup, we calculated the sizes of the different cavities in the membrane region present in the homology model. Within the expected errors related to model generation and considering that the main chain is likely more accurately assigned in the homology model than the side chains, the size of the cavity predicted with the highest confidence was comparable to that of the choline group of a PC molecule (ca. 99 Å^3^ compared to a volume of 119 Å^3^ for the choline group). Furthermore, it was possible to dock a phosphocholine headgroup within this cavity *in silico*. The headgroup is embedded in a deep central cavity delimited by TM3 and TM5 on the sides, with TM4 forming the back wall (Fig. 1c-e). The position of this phosphocholine headgroup suggests that the two fatty acid chains will be in contact with the hydrophobic portion of the lipid bilayer during transport. However, due to the lack of a membrane bilayer surrounding our structural model, it was impossible to obtain reliable results from *in silico* docking of a whole phospholipid. Within the cavity, the pi-cloud of the aromatic side chain of F375 of TM4 provides a hydrophobic environment compatible with the three methyl groups of the choline headgroup of PC, the preferred substrate for ALA10. Embedded within the pocket, the main chain carbonyl oxygens of Y374 and F375 point into the central cavity (Fig. 1c-e), similar to the situation in the SERCA Ca^2+^-ATPase, in which TM4 also contributes to the binding of the transported cation through the main carbonyl oxygens of amino acid residues surrounding a conserved central proline residue (P377 in ALA10). These carbonyl oxygens could potentially be involved in interactions with the positively charged nitrogen atom in the choline headgroup. The internal surface of the central cavity closer to the cytosol contains a positive electrostatic potential that could help accommodate the negatively charged phosphate group present in the lipid headgroup (Fig. 1f). Taken together, our structural model suggests that residues in TM4, which are likely to be deeply embedded in the membranous region, could play a role in substrate recognition by forming part of a lipid coordinating central cavity.

### ALA10 transports sphingomyelin

**Figure 2:**
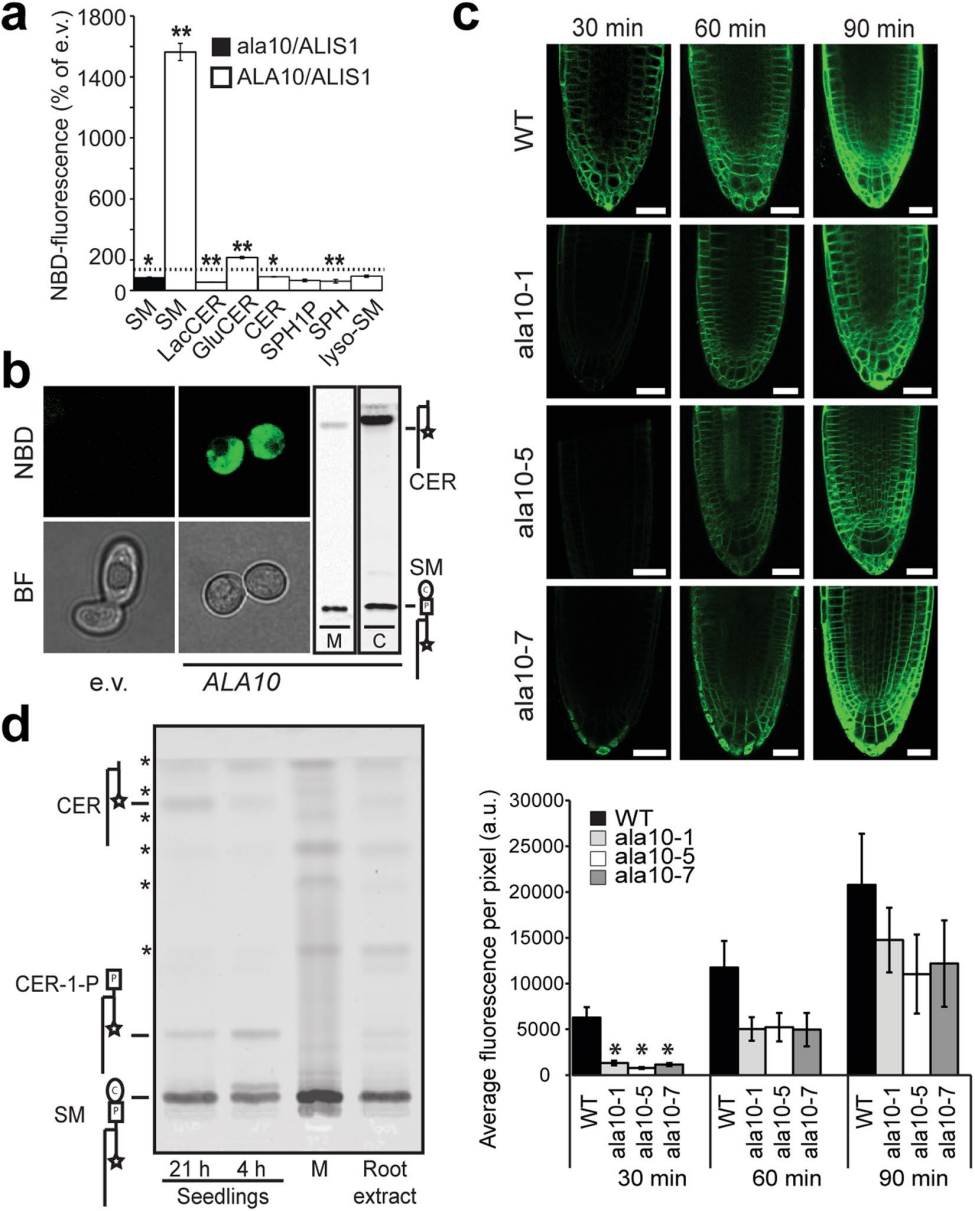
Sphingolipid internalization by ALA10/ALIS1 in yeast and planta. (**a,b**) *drs2*Δ*dnf1,2*Δ yeast cells expressing ALA10/ALIS1 or containing empty vectors were incubated with NBD-sphingolipids as indicated. (**a**) The fluorescence intensity of each cell was measured by flow cytometry and normalized to empty vector (e.v.) controls (set to 100%, dotted line). Catalytically inactive *ala10*D430N (*ala10*) was used as a control. Values are average ± s.e.m. of at least three experiments. **P < 0.005 and *P < 0.05, significantly different with respect to the e.v. control according to Student’s t-test. (**b**) Left: Cells were incubated with NBD-SM before imaging by fluorescence (NBD) or bright field (BF) microscopy. Scale bar, 5 µm. Right: Total lipid extraction and thin-layer chromatography were used to identify the lipid species present in the medium (M) and inside the cells (C). Medium and cells were run in parallel on independent TLC plates alongside lipid markers. (**c**) Five-day-old *Arabidopsis* seedlings from a wild-type (WT) Col-0 line or lines lacking ALA10 (*ala10*) were incubated in the presence of NBD-SM for the indicated periods, and visualized by confocal microscopy. Numbering of the *ala10* mutant lines indicates different alleles. Average pixel intensity measurements were used for quantification of fluorescent signals. *p < 0.05, significantly different with respect to wild type based on an ANOVA analysis. Data represent means ± s.d. (n = 5–7). Scale bar: 25 µm. (**b**) Wild-type Arabidopsis seedlings (Seedlings) or ground root tissue (Root extract) were incubated for 30 min in the presence of NBD-SM and washed with lipid-free media. Lipids were immediately extracted from root extracts or the incubation medium (M), before separation by thin-layer chromatography. Seedlings were allowed to rest for 4 or 21 h before lipid extraction. Lipid structures are given to the left (circle, choline; square, phosphate group; star, NBD group). Asterisks (*) indicate unidentified contaminants already present in the original NBD-lipid stock. Abbreviations: SM, sphingomyelin; LacCER, lactosylceramide; GluCER, glucosylceramide; CER, ceramide; SPH1P, sphingosine-1-phosphate; SPH, sphingosine; lyso-SM, lyso-sphingomyelin. Original scans for TLC plates can be found in Supplemental Datasets S2 and S3.

To test the limits of substrate recognition by ALA10, we aimed to determine whether this P4 ATPase also recognizes sphingolipids. For this purpose, we co-expressed ALA10 and its β-subunit ALIS1 in a *Saccharomyces cerevisiae drs*2Δ*dnf1,2*Δ triple mutant with negligible background flippase activity^16^. Analysis of NBD-lipid uptake by these cells revealed that the ALA10/ALIS1 complex is able to internalize SM labeled with the fluorescent probe 7-nitrobenz-2-oxa-1,3-diazole (NBD) and minute amounts of NBD-glucosyl ceramide, but not any other sphingolipid tested, including NBD-ceramide (NBD-CER; Fig. 2a). Confocal microscopy analysis confirmed that NBD-SM was indeed internalized, as the fluorescent signal can be visualized in internal membranes (Fig. 2b). Furthermore, a non-functional version of the transporter (*ala10*D430N), in combination with ALIS1, was unable to support the uptake of fluorescent SM, confirming that P4 ATPase activity is a requirement for lipid internalization (Fig. 2a). Thin-layer chromatography analysis of lipid extracts prepared from labeled cells and incubation media demonstrated that intact NBD-SM is readily internalized and then partially converted to NBD-CER (Fig. 2b).

ALA10 is expressed at the plasma membrane of root tip epidermal cells in *A. thaliana*, where it can support uptake of exogenously supplied fluorescently labelled lipids^12^. Taking advantage of this, the ability of ALA10 to flip NBD-SM was confirmed *in planta* through lipid uptake assays carried out on *A. thaliana* wild type (WT) and mutant lines lacking ALA10. Confocal microscopy visualization of root tips incubated with NBD-SM for different time periods demonstrated that accumulation of fluorescence in internal membranes is delayed in *ala10* knock-out plants (Fig. 2c). As for yeast, thin-layer chromatography analysis of lipid extracts demonstrated internalization of intact NBD-SM and partial conversion to NBD-CER (Fig. 2d). Based on these results, we conclude that the broad lipid specificity of ALA10 extends from glycerophospholipids to the sphingophospholipid SM.

### TM4 is involved in determining lipid specificity in ALA10

**Figure 3:**
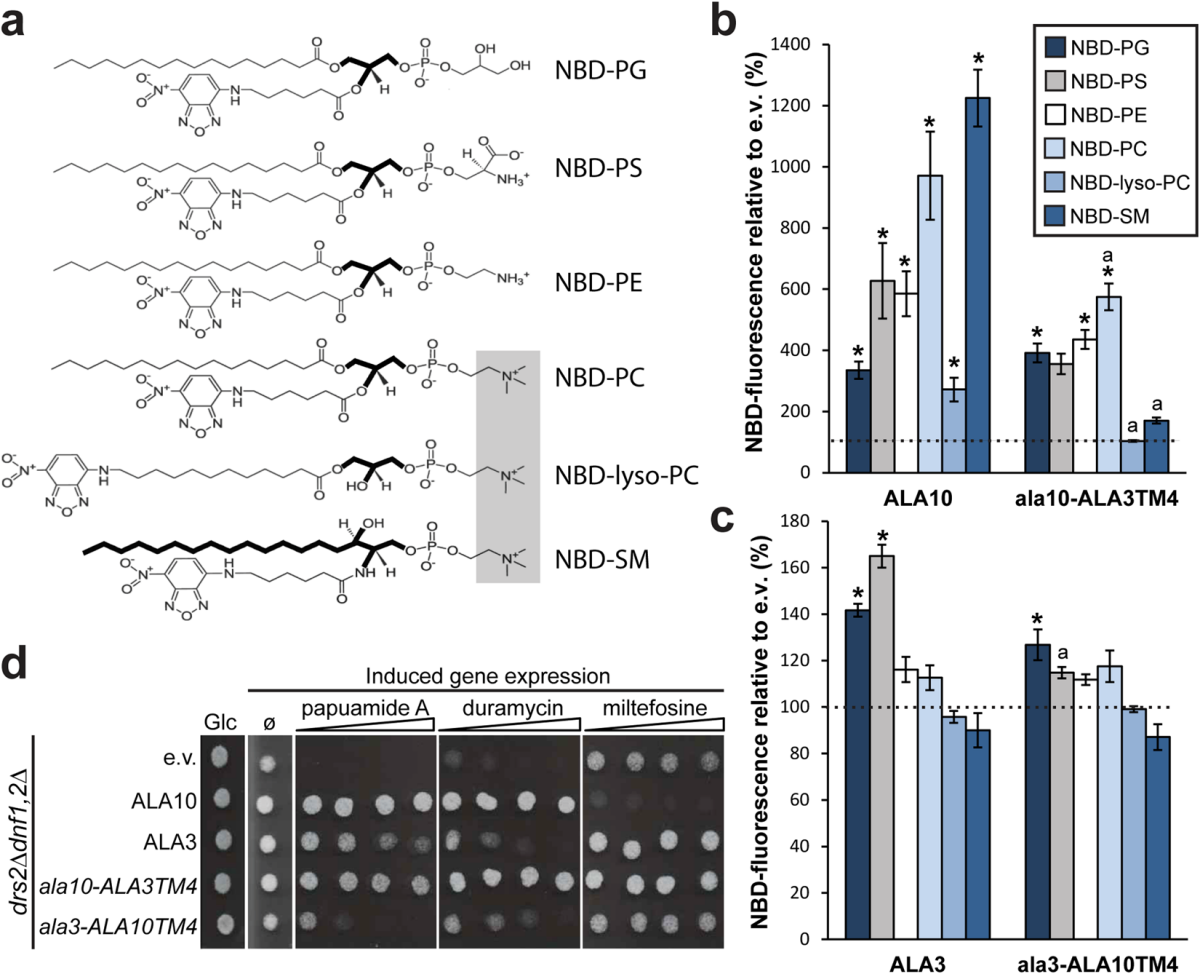
TM4 plays a role in determining the lipid specificity of ALA10 and ALA3. Chimeras of ALA10 and ALA3 in which TM4 was swapped between the proteins were generated, expressed in *drs2*Δ*dnf1*,*2*Δ yeast cells together with ALIS5, and characterized with respect to their lipid transport specificities using flow cytometry. WT ALA10 and ALA3 were used as controls. (**a**) Structures of the six NBD-lipids used in this study. The choline headgroup is shadowed gray and the glycerol and ceramide backbones are outlined in bold traces. (**b-c**) Accumulation of NBD-lipids by *drs*2Δ*dnf1*,*2*Δ cells transformed with *ala10*-ALA3TM4 (**b**) or *ala3*-ALA10TM4 (**c**) chimeras and ALIS5 shown as percentage of fluorescence intensity relative to *drs*2Δ*dnf1*,2Δ cells harboring empty vectors (e.v). Control levels are indicated by a dotted line. Results are averages ± s.e.m. from at least three independent experiments. Data were subjected to a two-factor ANOVA analysis of variance followed by Tukey’s HSD test: *p < 0.05, significantly different with respect to the empty vector control; a, significantly different (p<0.05) from ALA10 (**b**) or ALA3 (**c**) transport. (**d**) *drs*2Δ*dnf1*,2Δ yeast cells transformed with ALA10, ALA3, *ala10*-ALA3TM4, or *ala3*-ALA10TM4 together with ALIS5 were dropped onto a control plate with glucose (Glc) and onto galactose plates (Induced gene expression) with no further additions (ø) or containing a concentration gradient of the indicated toxins (direction of the gradient is indicated by a triangle). Cells transformed with empty vectors (e.v) served as a negative control.

To test the hypothesis that TM4 of ALA10 is involved in dictating its extraordinary substrate specificity, we swapped this transmembrane segment with that of another plant P4 ATPase, ALA3, which has previously been shown to have a preference for PS, and which is unable to transport SM^12,17,18^. Two chimeras were produced: *ala1*0-ALA3TM4, in which TM4 of ALA3 replaced the corresponding transmembrane segment in ALA10, and *ala3*-ALA10TM4, in which TM4 of ALA10 replaced TM4 of ALA3. Both chimeric proteins were co-expressed in yeast with the β-subunit ALIS5, which is known to be interchangeable with ALIS1^18^, and uptake of six different NBD-labeled phospholipids by the chimeric proteins was assayed (Fig. 3a). NBD-fluorescence intensities were expressed as percentages relative to control *drs2*Δ*dnf1,2*Δ cells harboring empty vectors (Fig. 3b and c). The preferred phospholipid substrates of ALA10 were SM > PC > PE/PS > PG, whereas those of the *ala10*-ALA3TM4 chimera were PC > PG/PE/PS > SM (Fig. 3b). Analysis of the data indicated that the *ala10*-ALA3TM4 chimera transported less NBD-PC than ALA10, while transport of NBD-PS, -PE and -PG were not significantly different from the wildtype. Furthermore, in contrast to ALA10, the *ala10*-ALA3TM4 chimera had completely lost the ability to transport NBD-lyso-PC and -SM.

With respect to empty vector controls, the *ala3*-ALA10TM4 chimera showed a low, but still statistically significant transport activity of NBD-PG, similar to ALA3 (Fig. 3c). Interestingly, in contrast to WT ALA3, NBD-PS transport was reduced in the *ala3*-ALA10TM4 chimera to levels not distinguishable from the empty vector controls, confirming that TM4 is important for selection of the transported lipid substrate.

As a complementary approach, we tested the effect of increasing concentrations of toxic peptides that bind to surface-exposed lipids on the survival of yeast cells containing the different ALA proteins. The lipid uptake defect of *drs2*Δ*dnf1,2*Δ yeast cells results in PS and PE being exposed to the outer leaflet of the plasma membrane, which renders the cells sensitive to the lipid-binding cytotoxic peptides papuamide A and duramycin, respectively^18^. Yeast *drs2*Δ*dnf1,2*Δ cells expressing ALA10 and* ala10*-ALA3TM4 survived similar concentrations of papuamide A and duramycin, suggesting that both proteins transported PS and PE. However, cells expressing the *ala10* chimera were less sensitive to high concentrations of miltefosine, a toxic analogue of lyso-PC, confirming that the chimera had lost its ability to transport lyso-PC (Fig. 3d). Also in accordance with our lipid transport results, *drs2*Δ*dnf1,2*Δ cells expressing ALA3 and the *ala3*-ALA10TM4 chimera survived similar concentrations of duramycin, while the WT protein was resistant to higher papuamide concentrations, confirming that the chimera is a less effective PS transporter.

### Residues Y374 and F375 located in the center of TM4 are critical for lipid specificity

**Figure 4:**
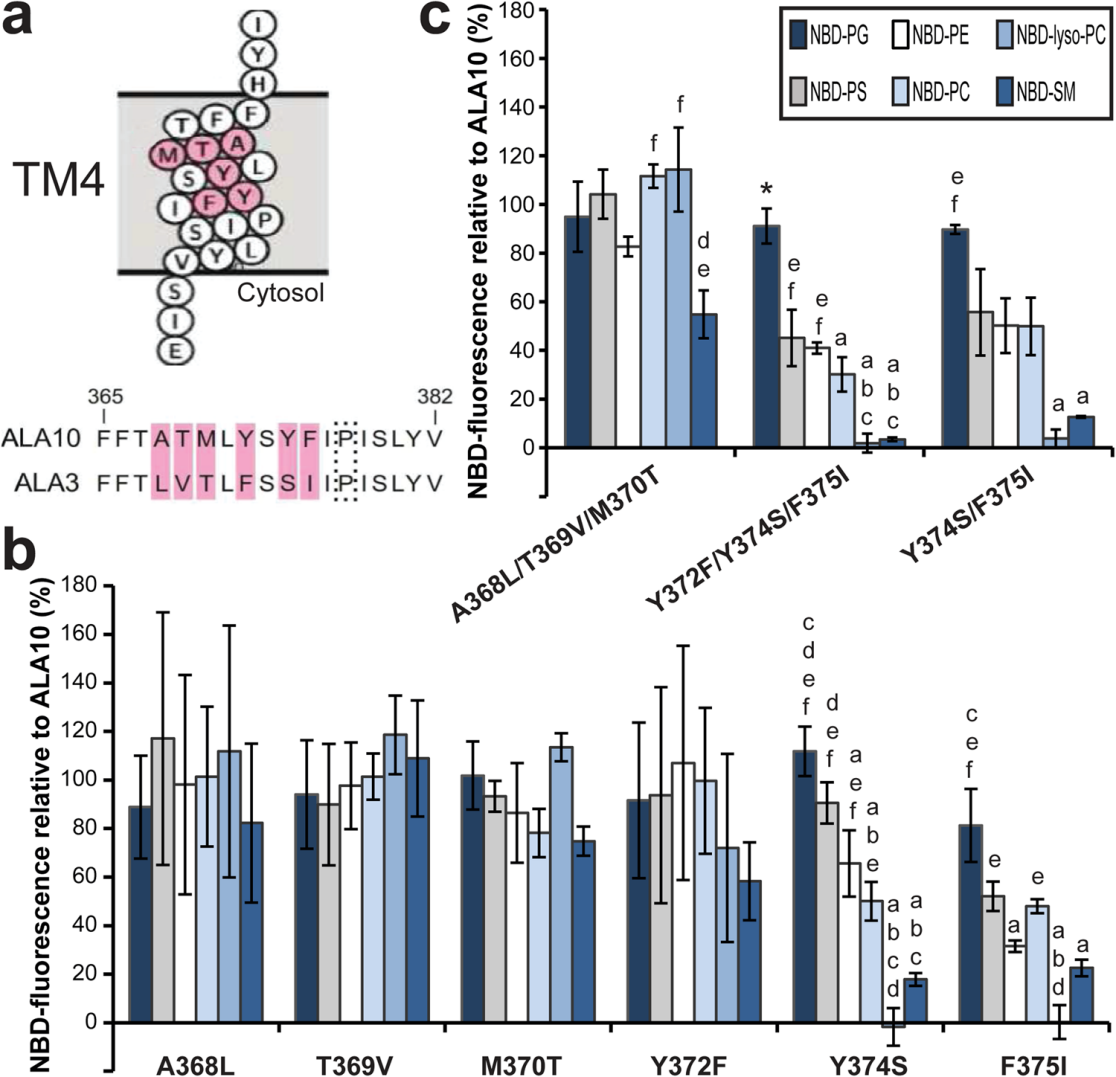
Residues located in the center of TM4 are involved in ALA10 lipid specificity. (**a**) Alignment of the TM4 sequences of ALA10 and ALA3, which were switched between the proteins to generate the chimeras *ala10*-ALA3TM4 and *ala3*-ALA10TM4. Residues differing between ALA10 and ALA3 are highlighted in pink. All six residues are located towards the center of the membrane within the ALA10 TM4 topology predicted by Protter and visualized by proteoform^28^. The conserved proline residue that unwinds the center of TM4 is marked by a dotted box. (**b**) and (**c**) ALA10 TM4 amino acid residues were mutated to the corresponding residues in ALA3 either individually (**b**) or in combination (**c**). Accumulation of NBD-lipids by *drs2*Δ*dnf1*,*2*Δ cells transformed with *ala10* point mutants and ALIS5 is shown as relative fluorescence intensity (%) with respect to *drs2*Δ*dnf1*,*2*Δ cells transformed with WT ALA10 and ALIS5. Fluorescence intensities with respect to control cells transformed with empty vectors are shown in Supplementary Fig. S2. Results are averages ± s.e.m. from at least three independent experiments. A Two-factor ANOVA followed by a Tukey’s HSD test was used for statistical analysis; a p value < 0.05 was considered significant. *: significantly different from all other lipids, a: significantly different from NBD-PG, b: significantly different from NBD-PS, c: significantly different from NBD-PE, d: significantly different from NBD-PC, e: significantly different from NBD-lyso-PC, f: significantly different from NBD-SM.

An alignment of TM4 in ALA10 and ALA3 revealed six residues that differ between the two proteins (Fig. 4a). Both proteins have an identical amino acid sequence after the conserved proline residue towards the cytosolic side of the membrane, suggesting that none of these residues are a determinant for specific lipid recognition. We next generated point mutations on ALA10 that changed each of the six identified residues to the corresponding ALA3 residue (A368L, T369V, M370T, Y372F, Y374S, and F375I). Only two mutants, *ala10*Y374S and *ala10*F375I showed significant changes in transport of several phospholipids with respect to wildtype ALA10 (Supplementary Fig. S2). In order to identify changes in lipid specificity, the NBD-fluorescence intensity values obtained for each mutant and each lipid were expressed relative to the corresponding value for WT ALA10 (Fig. 4b). The advantage of such an approach is that it allows correcting for possible differences in protein expression and/or trafficking to the plasma membrane^6,9^. In this type of representation, a change in lipid specificity for an *ala10* mutant with respect to the corresponding WT pump is observed as an increase or decrease in the relative fluorescence for a specific NBD-lipid with respect to the other NBD-lipids. Without a change in specificity the NBD-fluorescence for all lipids will decrease or increase to the same level with respect to WT transport.

A switch in lipid specificity was only observed for *ala10*Y374S and *ala10*F375I (Fig. 4b). Both point mutants displayed a significantly lower relative fluorescence for NBD-SM than for NBD-PG. In addition, both mutants had lost the ability to transport lyso-PC.

To confirm that Y374 and F375 are the only residues in TM4 responsible for the differences in specificity between ALA3 and ALA10, we generated double and triple *ala10* mutants and analyzed their lipid recognition pattern. First, we mutated A368, T369, and M370, located closest to the extracellular/lumenal side of TM4, to the corresponding ALA3 residues. No significant differences in lipid transport with respect to the wildtype or in specificity could be found for the triple* ala10*A368L/T369V/M370T mutant, except for a lower relative fluorescence of NBD-SM compared to NBD-PC, and -lyso-PC, but not to -PG, -PS or -PE (Fig. 4c and Supplementary Fig. S2). In contrast, mutation of the three amino acid residues closer to the conserved proline (*ala10*Y372F/Y374S/F375I) resulted in loss of NBD-lyso-PC uptake and a drastic reduction in NBD-SM transport, similar to the lipid recognition of the *ala10*-ALA3TM4 chimera. In addition, the triple *ala10*Y372F/Y374S/F375I mutant also transported NBD-PS and -PE to lower levels than the wildtype. A comparable result was obtained for a double *ala10*Y374S/F375I mutant, confirming that Y374 and F375 in TM4 have an important role in determining the lipid specificity of ALA10.

### The side chains Y374 and F375 in TM4 do not directly determine lipid specificity

**Figure 5:**
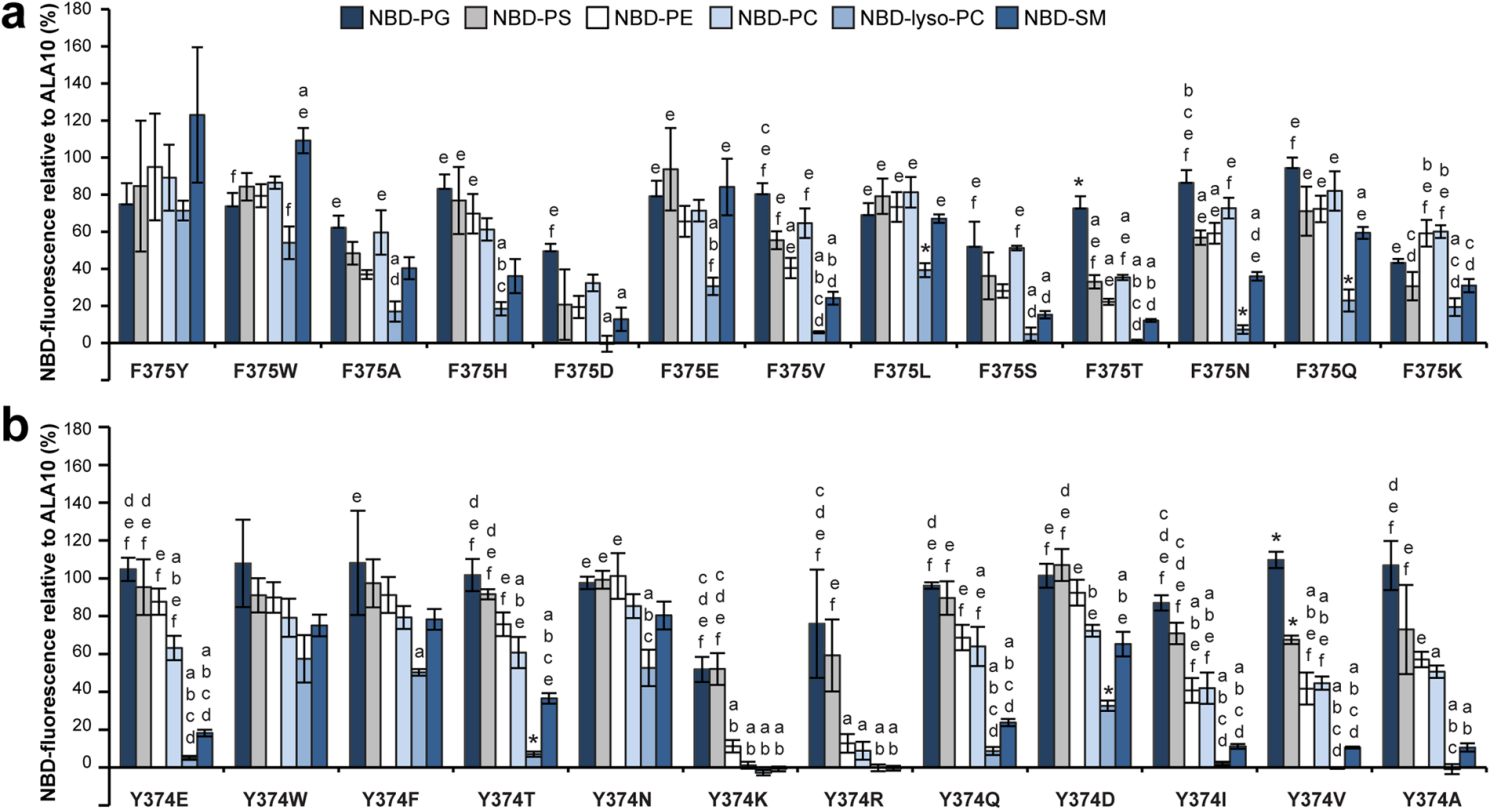
Changes in the side chain of the amino acid residues at positions 374 and 375 affect ALA10 lipid specificity. Accumulation of NBD-lipids by *drs2Δdnf1,2Δ* cells transformed with *ala10* point mutants in F375 (**a**) or Y374 (**b**), as indicated, and ALIS5 is shown as relative fluorescence intensity with respect to *drs2Δdnf1,2Δ* cells transformed with WT ALA10 and ALIS5. Results are averages ± s.e.m. from at least three independent experiments. A Two-factor ANOVA followed by a Tukey’s HSD test was used for statistical analysis; a pvalue < 0.05 was considered significant. *: significantly different from all other lipids, a: significantly different from NBD-PG, b: significantly different from NBD-PS, c: significantly different from NBD-PE, d: significantly different from NBD-PC, e: significantly different from NBD-lyso-PC, f: significantly different from NBD-SM.

According to our homology model, Y374 and F375, found to be required for the ability of ALA10 to recognize SM, have their carbonyl oxygens directed to the central cavity formed by TM3, TM4, and TM5 (Fig. 1c-e), where they could be involved in coordination of the lipid substrate. To test for possible roles of the side chains at these positions, we mutated each individual residue to a number of other amino acids, with side chains of different polarities, lengths, and sizes (Fig. 5 and Supplementary Fig. S3).

Mutations F375W, F375Y, F375H, F375E did not show significant differences in lipid transport with respect to WT ALA10 (Supplementary Fig. S3) and only small specificity changes could be observed for these mutants (Fig. 5a). In contrast, mutations F375T, F375N, F375S and F375V affected recognition of almost all lipids. Only one mutant, F375K, seemed to transport all lipids to lower levels than wildtype ALA10, suggesting defective expression or trafficking to the plasma membrane. In order to further assess the changes in specificity for each individual mutant, the relative fluorescence with respect to WT ALA10 was calculated (Fig. 5a). While almost all mutations resulted in specificity changes, these could not be assigned to recognition of a specific lipid headgroup, backbone, or acyl chain number. For instance, for F375A the relative fluorescence values for NBD-PG, -PS, -PE, -PC and -SM, bearing different backbones and lipid headgroups, were not significantly different. In contrast, the relative fluorescence for NBD-lyso-PC, bearing only one fatty acid chain, was significantly different from NBD-PG and -PC, but not -PS, -PE and -SM, all of which carry two fatty acid chains. For a F375S mutant, the relative fluorescence for NBD-PC is significantly different from -lyso-PC and -SM, even when these lipids bear the same phosphocholine headgroup, suggesting that the differences might be related to recognition of the backbone or the fatty acyl chain number. However, no significant differences could be found between NBD-PS or -PE (bearing the same backbone and fatty acyl chain number as PC), with respect to -lyso-PC or -SM.

Similarly, mutations Y374W and Y374F resulted in lower transport of NBD-lyso-PC and -SM with respect to the wildtype, while a Y374N mutant transports -lyso-PC less efficiently than ALA10 (Supplementary Fig. S3). In contrast, mutations Y374K, Y374R and Y374V transport all lipids to lower levels than the wildtype, except for NBD-PG. Only one mutation, Y347I seemed to affect transport of all lipids, suggesting that this mutant might be expressed to lower levels than the wildtype at the plasma membrane. Interestingly, changes of Y374 to a positively charged amino acid (Y374K and Y374R) seemed to specifically favor transport of negatively charged lipids (NBD-PG and -PS), almost totally eliminating transport of other lipids (Fig. 5b and Supplementary Fig. 3). Notably, mutations that introduce a neutral side chain (Y374I and Y374V) also reduced transport of phospholipids with a neutral headgroup to a bigger extent than for negatively charged lipids, although these mutants still showed a preference for PC as a substrate. For all other mutants, the differences in specificity could not be assigned to a concrete lipid headgroup, backbone, or acyl chain number (Fig. 5b).

Taken together, these results are compatible with a model in which the side chains of Y374 and F375 are not directly binding the lipid substrate, but are important for controlling lipid specificity, probably by generating an appropriate environment for the lipid headgroup through the side chain of Y374 and by changing the dimensions of the coordinating cavity, as suggested by our homology model.

### Substitutions of I378 still allow for lipid translocation

**Figure 6:**
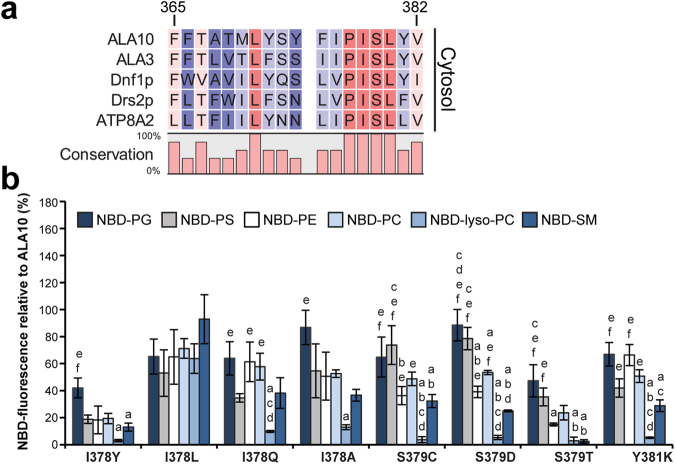
Changes in the side chain of the amino acid residues located after a conserved proline in TM4 affect ALA10 lipid specificity. (**a**) Alignment of predicted transmembrane domain 4 sequences for selected P4 ATPases from *Saccharomyces cerevisiae* (Dnf1p, Drs2p), *Arabidopsis thaliana* (ALA10, ALA3) and *Bos taurus* (ATP8A2) showing the strict conservation of amino acid residues towards the cytosolic side. Numbers correspond to ALA10 residues. (**b**) Accumulation of NBD-lipids by *drs2Δdnf1,2Δ* cells transformed with the indicated *ala10* point mutants and ALIS5 is shown as relative fluorescence intensity with respect to *drs2Δdnf1,2Δ* cells transformed with WT ALA10 and ALIS5. Results are averages ± s.e.m. from at least three independent experiments. A Two-factor ANOVA followed by a Tukey’s HSD test was used for statistical analysis; a p value < 0.05 was considered significant. *: significantly different from all other lipids, a: significantly different from NBD-PG, b: significantly different from NBD-PS, c: significantly different from NBD-PE, d: significantly different from NBD-PC, e: significantly different from NBD-lyso-PC, f: significantly different from NBD-SM.

While the amino acid residues located to the extracytosolic side of TM4 in P4 ATPases show a very low degree of conservation, the residues located to the cytosolic side of the conserved proline are almost strictly conserved among P4 ATPases from different organisms (Fig. 6a and Supplementary Dataset S1). Mutagenesis studies in the mammalian P4 ATPase ATP8A2 suggested that these conserved residues, especially I364 (located right beside the conserved proline), form a hydrophobic gate separating two water-filled cavities that would allow entry and exit of the lipid headgroup^10^ (Fig. 1a). In our model, the side chain of I378 in ALA10 (corresponding to I364 in ATP8A2) points away from the putative lipid coordinating cavity, and is unlikely to be involved in direct gating of lipid transport. However, changes in this side chain might affect the position of the unwound segment of TM4 and the dimensions of the cavity, thus affecting lipid specificity. In order to test this, we generated *ala10* mutants bearing a conservative substitution of I378 (I378L) or substitutions to a bulky aromatic amino acid (Y), a small amino acid (A), or an amide (Q) amino acid (Fig. 6b and Supplementary Fig. S4a). Despite the drastic differences between the side chains, all generated mutants supported lipid translocation at the plasma membrane to some extent (Supplementary Fig. S4a). A conservative change (I378L) did not affect lipid specificity (Fig. 6b). Slight specificity changes were observed for the I378Y, I378Q and I378A mutants, although these could not be assigned to recognition of a specific lipid headgroup, backbone, or acyl chain number. These results suggest that the side chain of I378 might be important to control the dimensions of the lipid coordinating cavity, as suggested by the homology model.

### Substitution of S379, but not Y381, specifically affects transport of lipids with a neutral headgroup

Another two amino acid residues on the cytosolic side of TM4 have been suggested to affect the kinetic properties of ATP8A2^10^: S365 (at position +2 with respect to the conserved proline, Fig. 6a), which is conserved in plants, yeast and mammalian flippases, and L367 (P + 4 position), which corresponds to a tyrosine (Y618) that has been implicated in selection against PS in yeast Dnf1p. Our model predicts that the side chain of S379 (corresponding to S365 in ATP8A2) is pointing away from the lipid-coordinating cavity and presumably contributing to the positioning of TM4 during transport. Mutations S379C and S379D showed a drastic reduction in the relative fluorescence for NBD-lyso-PC with respect to all diacylglycerophospholipids (NBD-PG, -PS, -PE and -PC), but not with respect to NBD-SM (Fig. 6b and Supplementary Fig. S4a). In contrast, a S379T mutation almost completely abolished transport of both NBD-lyso-PC and -SM. A S379Y mutant was still expressed in yeast and reached the plasma membrane (Supplementary Fig. S4b). However, no activity could be detected for this mutant (Supplementary Fig. 4a), suggesting that the bulkiness of the side chain might be relevant for lipid translocation.

Mutation in an amino acid residue located closer to the cytosolic side of the membrane, Y381K (corresponding to L367 in ATP8A2 and Y618 in Dnf1p), resulted in a preference of the mutant protein for diacylglycerophospholipids (NBD-PG, -PS, -PE, -PC) with respect to monoacylated lyso-PC, in analogy to the S379C and S379D mutants (Fig. 6b). Taken together, these results suggest that the side chains of S379 and Y381 are important for defining the position of the unwound part of TM4, thereby helping define the dimensions of the lipid-coordinating cavity.

## Discussion

Our homology model for ALA10 suggests the presence of an elongated cavity in the middle of the membrane region that is flanked by TM3, TM4, and TM5. The size of this groove and its positive electrostatic surface potential (Fig. 1f) make it a strong candidate region in the transmembrane domain that could accommodate a phospholipid headgroup and neutralize the negative charge of its phosphate moiety. In accordance, we successfully docked a phosphocholine headgroup (the preferred substrate for ALA10) in this central cavity. A crystal structure of the sarcoplasmic reticulum Ca^2+^-ATPase SERCA in the E2 conformation^19^ (pdb ID: 3AR4) shows the inhibitor thapsigargin bound at a cleft corresponding to the groove observed in our homology model. This may suggest that a groove between TM3, TM4, and TM5 is conserved among P-type ATPases. Indeed, the position of thapsigargin in this structure coincides with that of the docked phosphocholine headgroup in our homology model (Supplementary Fig. S5).

Docking of the phosphocholine headgroup into the central cavity also showed that only the side chain of F375 is at a position where it might interact with the lipid headgroup by using its pi-cloud to coordinate the headgroup methyl groups, which could account for the broad lipid specificity exhibited by ALA10. Moreover, the position of the headgroup in the cavity would leave the lipid backbone outside the protein in contact with the membrane, thus explaining why ALA10 recognizes ceramide-based NBD-SM. While SM is only a naturally occurring lipid in mammals, none of the mammalian P4 ATPases characterized so far have been shown to transport SM. In WT yeast, uptake of NBD-SM can be measured under certain conditions^20^. However, this transport is not dependent on ATP, and was assigned to passive diffusion events. Mutation of a residue on the cytosolic flanking region of TM1 in yeast Dnf1p (N220S) confers the protein the ability to recognize SM^21^. However, this asparagine is highly conserved among P4 ATPases, including ALA10, suggesting that this residue is not a natural determinant of substrate recognition, or that a specific combination of residues from different TM segments is necessary to allow SM recognition.

An analysis of a homology model based on the crystal structure of SERCA2 suggested that the yeast plasma membrane P4 ATPase Dnf1p, which transports PC and PE, has a groove between TM1 and TM3, with TM4 forming the back wall^6^. In that work, mutation of a tyrosine (Y618) to phenylalanine in TM4 (corresponding to Y381 in ALA10), which has its side chain directed into the proposed groove, generated a change in specificity of Dnf1p towards PS. By contrast, mutating this residue to leucine did not change the specificity. Here we demonstrate that this tyrosine in ALA10 TM4 is indeed important for lipid translocation, as a mutation Y381K affects lipid transport, but is not a key determinant for recognizing the serine headgroup or the PS negative charge. Interestingly, another groove is present in the Dnf1p structural model between TM3, TM4, and TM5^6^. In analogy to our structural model, the carbonyl oxygens of the main chain of Q610 and S611 (corresponding to Y374 and F375 in ALA10) are directed to the inside of the cavity. The possible role of this groove in lipid translocation was not analyzed, as the exchange of TM5 between Dnf1p and its PS-transporting relative Drs2p resulted in an inactive protein. Our model suggests that this inactivation might be due to a change in the dimensions of the gap between TM3 and TM5, which would interfere with loading of the lipid substrate. Furthermore, point mutations in several residues in TM5 and TM6 of Dnf1p result in a reduced protein activity without changing the lipid recognition pattern of the protein. Thus these two TMs might have a role in lipid coordination that does not involve direct binding of the lipid headgroup, and which could be explained by the position of these TMs with respect to the lipid-coordinating central cavity present in our homology model.

The Dnf1p model led to the proposal of a two-gate mechanism in which the lipid is recognized at both an entry gate and an exit gate^6,8,9^. The entry gate is composed of residues clustering at the edge of the extracellular/lumenal side of the membrane and in the extracellular loops between TM1 and TM2, and TM3 and TM4. The exit gate consists of residues of TM1, TM2, TM3, and TM4 at the cytosolic edge of the membrane. In our homology model, residues located in the middle of TM1 and TM2 are not expected to participate directly in the central cavity; however, mutation of these residues could have indirect effects on the central cavity by altering the transmembrane dynamics. Notably, TM1 and TM2 are positioned right behind and close to TM4 in our model. Therefore, it is possible that some residues in the middle of TM4 interact directly with residues in TM1 and TM2.

A second model, the ‘hydrophobic-gate’ mechanism, for P4 ATPase action was proposed for mammalian PS-transporting ATP8A2, which also shows background activity for PE^22^. According to this model, a groove is present between TM1, TM2, TM4, and TM6, and the distance between TM1 and TM2 and the other TM domains is considerably larger than in the crystalized P-type ATPases. This separation generates two water-filled cavities, with alternate access to the cytosolic or the extracellular/lumenal side, respectively. In our model, such a large separation between transmembrane domains is not present (Supplementary Fig. S6). In ATP8A2, I364 in TM4 was shown to be important for ATPase activity, lipid translocation, and lipid affinity^10^. These results are compatible with our model, in which the side chain of I378 (the corresponding residue in ALA10) points towards the space between TM1 and TM2. Our results suggest that mutation of the side chain of I378, or its adjacent residue S379, would likely displace the unwound part of TM4 and change the dimensions of the proposed phospholipid headgroup-coordinating cavity. Moreover, previous evidence suggested that a conserved lysine in TM5 of ATP8A2 was essential for lipid translocation^7^, but none of the available models can account for this fact. Interestingly, in our model, the side chain of this lysine (K929 in ALA10) points away from the cavity into the space between TM6 and TM8, and could establish a hydrogen bridge with the hydroxyl group of Y374 in TM4, which points towards TM6. Notably, all residues in TM6 and TM8 that are close to the side chain of K929 are aromatic or hydrophobic (Supplementary Fig. S7).

The homology model of ALA10 revealed a central cavity between TM3, TM4, and TM5, in which the main chain carbonyl oxygens of both Y374 and F375 (located on the extracytosolic side of the conserved proline in TM4) are available for coordination of the lipid headgroup and/or backbone (Fig. 1). Although our results are compatible with coordination of the lipid into the cavity through these carbonyl oxygens, the observed changes in lipid specificity for the different *ala10* mutants could have two different causes. On the one hand, they could derive from changes in the affinity for the lipid substrate, which would support direct involvement of Y374 and F375 in headgroup coordination. On the other hand, they could be caused by a change in Vmax, e.g. an slowing or acceleration of a rate-limiting conformational transition during the catalytic cycle, which might be dependent on the exact position of TM4. Notably, mutation of residues located on the cytosolic side of the conserved proline (I378, S379, Y381) also results in changes in lipid specificity that are compatible with these residues affecting the position of TM4. Therefore, we cannot rule out that Y374 and F375 are also affecting the position of this TM and are not directly involved in lipid coordination through their carbonyl oxygens. Resolving these two possibilities would require biochemical assays on purified proteins. Unfortunately, the low level of expression of plant P4 ATPases in yeast hampers such an approach.

The central cavity lined with carbonyl groups present in our model resembles the cavity in the plasma membrane H^+^-ATPase^14^. Indeed, in this structure, the main chain carbonyl groups of TM4 residues, close to the conserved proline, are exposed to the binding cavity. Furthermore, a mutagenesis study of TM4 residue I282 of the plasma membrane H^+^-ATPase demonstrated the effects on H^+^ transport that were related to the size but not the chemical properties of the side chain at this position^14,23^. This was taken as evidence that the main chain carbonyl oxygen of this residue plays a role in transporting protons.

While the spacious gap between TM3 and TM5 provides direct access to the cavity, the aromatic side chain of F375 pointing out between TM3 and TM5 could function as a gate-keeper in the E2P-state, keeping the lipid from flipping back towards the extracellular/lumenal side of the membrane. This is supported by the results showing that removing the aromatic side chain at this position alters the lipid specificity of ALA10, but does not prevent lipid translocation (Fig. 4), suggesting that this side chain has a role independent of lipid headgroup coordination. Furthermore, the flexibility of TM4 originating from the unwound center could facilitate the movement of the F375 side chain away from the TM3/TM5 groove in the E1P-state, providing open access to the cavity for lipid loading.

The transmembrane domain cavity observed in the central cavity model presented herein provides a spacious access groove between TM3 and TM5 (Fig. 1). This would allow the headgroup of the large lipid substrate to be coordinated in the central cavity, while the fatty acid tails remain in contact with the hydrophobic membrane environment. Given the broad lipid specificity exhibited by ALA10, further work is required to demonstrate whether this model can be applied to other much more selective P4 ATPases.

## Methods

Full methods are described in Supplementary Methods. The primers, templates, and plasmids used in this study are listed in Supplementary Tables S1 and S2.

### Homology modelling and docking

A multiple sequence alignment including around 50 randomly (and non-redundant) selected members of P-type ATPase classes 1B, 2A, 2C, 3A and 4, characterized by their different transport specificities, was generated using MUSCLE software and revised manually. A truncated version of this alignment (Supplementary Dataset S1) was used to generate an ALA10 homology model using MODELLER software. All the selected crystal structures corresponded to transition states of dephopshorylation (E2∙P_i_) in between the E2P and E2 intermediates of the E1—E1P—E2P—E2 P-type ATPase reaction cycle. Images were generated using PyMOL (http://pymol.sourceforge.net). Electrostatic potential was calculated using the APBS plugin. Docking calculations were performed with the docking program AutoDock Vina^24^ after preparation of the ALA10 homology model and the ligand Dock Prep^25^ in UCSF Chimera^26^, using standard settings.

### Yeast strain and culture

*S. cerevisiae* mutant strain ZHY709 (*MATα his3 leu2 ura3 met15 dnf1Δ dnf2Δ drs2::LEU2)* ^16^ was used and transformed by the lithium acetate method. Transformants were cultured at 30 °C in standard synthetic glucose (SD) or galactose (SG) medium lacking the appropriate amino acids. SG gradient plates were stored at 4 °C for 2 days before use. Gradients contained the following maximum concentrations: 0.3 µg/mL papuamide A (Flintbox, Lynsey Huxham), 3 µM duramycin (Sigma-Aldrich), or 5 µg/mL miltefosine (hexadecylphosphocholine, Calbiochem, La Jolla, CA).

### NBD-lipid uptake assays, microscopy, and lipid analysis

Fluorescent NBD-lipids were purchased from Avanti Polar Lipids (Birmingham, AL, USA) and stocks were prepared in DMSO. Uptake experiments in yeast were performed essentially as described previously^12^ (See also Supplemental Methods). Flow cytometry was performed on a Becton Dickinson Flow Cytometer equipped with an argon laser using Cell Quest software. Data were analyzed using Cyflogic (CyFlo, Ltd), according to^27^. Data for at least 20,000 cells was collected prior to gating. NBD-fluorescence of living cells was plotted on a histogram and the geometric-mean fluorescence intensity was used for further statistical analysis. Lipid uptake assays in *Arabidopsis* were performed as described^12^. Briefly, plate-grown 5-day-old seedlings were incubated in ½ Murashige and Skoog (MS) liquid medium in the presence of 40 µM NBD-SM for the indicated periods, and washed twice with lipid-free medium before visualization. Fluorescence microscopy and image acquisition were carried out using a spectral confocal laser scanning microscope (Leica Microsystems, Heidelberg, Germany) equipped with a 100x/1.40 N.A. oil immersion objective (yeast) or 63x/1.0 N.A. objective (planta). NBD was excited at 458 nm and emission signals were recorded between 495 and 550 nm. All fluorescent signals were quantified using ImageJ (http://imagej.nih.gov/ij/). For analysis of lipid metabolism, lipids were extracted and separated by thin-layer chromatography as previously described^12^. Fluorescent lipid spots were visualized with a Typhoon Trio Variable Mode Imager (GE Healthcare, Brøndby, Denmark).

### Data analysis

Data were analyzed using Excel (Microsoft). Data represent means ± s.e.m. of at least 3 experiments. Statistical analysis of sphingolipid transport data with respect to empty vector controls in Fig. 2a was carried out using a Student’s t-test, with a two-tailed distribution and two-sample unequal variance. For lipid uptake assays in planta, a single-factor analysis of variance (ANOVA) followed by Dunnett’s test was performed independently for each time point. For lipid uptake assays in yeast (Figs 4-6), a background correction was made by subtracting the raw values of NBD-fluorescence obtained for empty vector controls from the corresponding values obtained for each *ala10* mutant and each lipid in the same experiment. Then, the corrected values for each mutant and lipid were normalized to the corresponding value for the wild-type version of ALA10, which was set to 100%. Statistics on transport rates with respect to wild-type proteins (Fig 3b and c and Supplementary Figs S2, S3 and S4) were performed using two-factor analysis of variance (ANOVA) with blocking followed by a Tukey’s honest significant difference (HSD) test.

### Data availability

Sequence alignment data used during this study is included in the Supplementary Information. Flow cytometry datasets generated for lipid transport assays and the ALA10 structural homology model can be downloaded from Figshare under doi 10.6084/m9.figshare.5441542.

## Electronic supplementary material

Supplementary Information
