# Supplementary material for: Phospholipid flipping involves a central cavity in P4 ATPases
Source: Sci Rep. 2017 Dec 15;7:17621. doi: 10.1038/s41598-017-17742-y (PMC5732287; doi:10.1038/s41598-017-17742-y)

# Supplementary information for

## Phospholipid flipping involves a central cavity in P4 ATPases

*Jensen MS, Costa S, Duelli AS, Andersen PA, Poulsen LR, Stanchev LD, Gourdon PE, Palmgren M, Pomorski TG, López-Marqués RL*

This file includes:

- Supplementary Methods
- Supplementary Figures S1-S7
- Supplementary Tables S1 and S2
- Supplementary Dataset S1: alignment used for the ALA10 structural model
- Supplementary Dataset S2: Original scans for the TLC plates used in Figure 2 at different exposure times
- Supplementary Dataset S3: Original scans for the TLC plates used in Supplementary Figure S2 at different exposure times.

**Supplementary Methods**

***Homology modelling***

The ALA10 homology model was generated using the MODELLER software (version 9.15), and based on the following crystal structures of P-type ATPase classes with different transport specificity: spiny dogfish $Na^+/K^+$-ATPase (pbd-id 2ZXE as an example of a class P2C P-type ATPase)[1] , the rabbit SERCA1a $Ca^{2+}$-ATPase (pbd-id 1WPG, class P2A)[2], the Legionella pneumophila lpg1024 $Cu^+$-ATPase (pbd-id: 4BYG, class P1B-1) and the Shigella sonnei $Zn^{2+}$-ATPase (pbd-id 4UMW, class P1B-2)[3]. All the selected structures correspond to transition intermediates of dephosphorylation (E2.$P_i$) in-between E2P and E2 in the E1-E1P-E2P-E2 P-type ATPase reaction cycle, suggesting that their overall fold is maintained (as also observed through structural alignment[4]). No proton ATPase was included during modelling, due to lack of an equivalent E2.$P_i$ structure. The built sequence alignment originally included around 50 randomly (and non-redundant) selected members of P-type ATPase classes 1B, 2A, 2C, 3A (proton pumps) and 4 (flippases), and was generated using the software Muscle (version 3.8.31)[5]. Correct alignment of transmembrane spanning helices for the different P-type ATPase classes was ascertained manually. Finally, the alignment was truncated to consist of ALA10, 1WPG, 2ZXE, 4BYG and 4UMW (see Supplementary Dataset S1). MODELLER then implemented comparative protein structure modeling by satisfaction of spatial restraints through an iterative process. Images were generated using PyMOL (http://pymol.sourceforge.net). Electrostatic potential was calculated using the APBS plugin.

***Docking experiments***

The docking calculations were performed with the docking program AutoDock Vina[6]. Prior to docking calculations the ALA10 homology model and the ligand were prepared with Dock Prep[7] in UCSF Chimera[8], using standard settings. The search space was included in a box of 20 x 20 x 20 Å centered on the amino acid residues Y374, F375 and P377. The calculation resulted in 7 possible orientations of the ligand in the binding pocket, with 4 orientations showing the phosphate head group pointing into the binding pocket. These 4 solutions had lower RMSD values (between 0 and 1.4) compared to the RMSD values of residual orientations (3.7-3.8).

***Cloning and plasmids***

Primers and templates used for PCR-based cloning are listed in Supplementary Table S1. Plasmids used in this study are listed in Supplementary Table S2. All PCR reactions were carried out using Phusion® High-Fidelity DNA Polymerase (New England Biolabs, Ipswich, USA), according to the manufacturer's instructions.

For expression of ALA10, a plasmid bearing the *ALA10* cDNA sequence fused to an N-terminal RGSH10 tag and under the control of a bi-directional GAL1-10 promoter was used (pMP3390)[9]. To generate an analogous ALA3 construct, the full cDNA for *ALA3* was PCR amplified from a previously described plasmid[10] using primers that introduce artificial *EcoR*I and *Apa*I sites at the 5'- and 3'-end, respectively. The PCR product was cloned into pCR™4 Blunt-TOPO® using the Zero Blunt-TOPO® PCR Cloning Kit for Sequencing (Invitrogen, Carlsbad, USA) according to manufacturer's instructions, to generate the pMP3163 plasmid. The *ALA3* sequence was excised from this plasmid by *EcoR*I/*Apa*I digestion and ligated into pMP4062[11] cut with the same enzymes, rendering the pMP4133 plasmid, which carries an RGSH10-ALA3 fusion under the control of the GAL1-10 promoter.

To generate point mutations in TM4 of ALA10 and the *ala10*-ALA3TM4 chimera (pMP4378), where TM4 of ALA10 was swapped for the TM4 of ALA3, a cloning strategy based on homologous recombination in yeast was used. In brief, TM switch and point mutations were introduced by two PCRs with an overlapping region, including the mutation, followed by transformation of *S. cerevisiae* strain ZHY709[12] together with an ALA10 containing plasmid (pMP3390) cut with *EcoR*I.

In the case of ala3-ALA10TM4 (pMP4883), an overlapping PCR strategy was used. In the first PCR round, two PCR products were obtained that correspond to i) the N-terminal region of ALA3 to the start of TM4 fused to the TM4 sequence of ALA10 and ii) TM4 for ALA10 fused to the sequence of ALA3 from the end of this transmembrane domain. A total of 0.5 µL of each PCR was then used as template for a second PCR round to amplify the full-length ALA3 chimera. The resulting PCR product and pMP4133 were digested with *EcoR*I and *Apa*I, followed by enzymatic ligation, yielding pMP4883.

For all PCRs performed to generate *ala10* point mutants, plasmid pMP3136 was used as template. Plasmid pMP3136 was generated by amplifying full-length *ALA10* (At3g25610) through overlapping PCR using purified root complementary DNA (cDNA) from *A. thaliana* Col-0 as template and oli_1938, oli_1939, oli_2042, and oli_2043. The PCR product was cloned into the pCR®4 Blunt-TOPO® vector using the Zero Blunt® TOPO® PCR Cloning Kit (Life Technologies, Carlsbad, CA, USA).

After homologous recombination, plasmids were isolated from yeast by lysing the cells with acid-washed 0.5 mm glass beads followed by purification of DNA using the GenElute™ Plasmid Miniprep Kit (Sigma-Aldrich, St. Louis, USA) and amplification in *E. coli*[13]. Correct vector construction was confirmed by DNA sequencing.

***Yeast strain and culture***

*S. cerevisiae* mutant strain ZHY709 (*MATα his3 leu2 ura3 met15 dnf1Δ dnf2Δ drs2::LEU2)*[12] was used and transformed by the lithium acetate method[14]. For co-expression of *ALA10* and *ala10* mutants with *ALIS5*, cells were simultaneously transformed with two individual plasmids bearing the desired genes and histidine or uracil auxotrophic markers. For growth assays, transformants were grown in 1 mL Synthetic Complete Galactose (SCG) medium (0.7% (w/v) Yeast Nitrogen Base, 2% (w/v) galactose supplemented with 1.4 g/L yeast synthetic dropout medium lacking histidine and uracil (Sigma-Aldrich, St. Louis, USA)) at 30°C with 150 r.p.m. shaking for 4 h to induce overexpression. Cultures were diluted with water to 0.1 $OD_{600}$/mL, and either 5 or 3 µLwas spotted onto solid SG (2% agar added) or synthetic complete glucose (SD) medium plates (same as SG but with 2% (w/v) glucose instead of galactose), and incubated for 4 days at 30°C. SG gradient plates were prepared as previously described[15] except that they were stored at 4°C for 2 days before use to allow for diffusion of the toxins. Gradients contained the following maximum concentrations: 0.3 µg/mL of papuamide A (Flintbox, Lynsey Huxham), 3 µM of duramycin (Sigma-Aldrich), or 5 µg/mL of miltefosine (hexadecylphosphocholine, Calbiochem, La Jolla, CA). All experiments were repeated independently at least three times. For NBD-lipid translocation assays, transformants were grown essentially as previously described[16].

**Plant growth conditions**

*A. thaliana* seeds corresponding to wild type Col-0, and mutant lines lacking *ALA10* (*ala10-1* (SALK_024877), *ala10-5* (WiscDsLox_499E11), or *ala10-7* (GABI_78G10)) were surface-sterilised and transferred to plates containing ½ Murashige and Skoog[63] (½MS) media including vitamins (Duchefa Biochemie; Amsterdam, The Netherlands), 0.5 g/L 2-(N-morpholino)ethanesulfonic acid (MES) and 1% agar (A1296; Sigma Aldrich, St. Louis, MO, USA), buffered to pH 5.7 by the addition of KOH. After a 3-day vernalisation, plates were transferred to a growth chamber and incubated under a 12-h light regime with the following scheme: t=0 h, 20°C, ≈40 µE light intensity; t=3 h, 20°C, ≈160 µmol photons $m^{-2}$ $s^{-1}$ light intensity; t=7 h, 22°C, ≈85 µmol photons $m^{-2}$ $s^{-1}$ light intensity; t=10 h, 20°C, ≈40 µmol photons $m^{-2}$ $s^{-1}$ light intensity; t=12 h, 20°C, darkness; t=16 h, 17°C, darkness; t=20 h, 15°C, darkness; and t=22 h, 17°C, darkness.

***NBD-lipid uptake assays***

Fluorescent 7-nitrobenz-2-oxa-1,3-diazole (NBD)-lipids, including palmitoyl-(NBD-hexanoyl)-PS (NBD-PS), palmitoyl-(NBD-hexanoyl)-PE (NBD-PE), palmitoyl-(NBD-hexanoyl)-PC (NBD-PC), palmitoyl -(NBD-hexanoyl)-phosphatidylglycerol (NBD-PG), NBD-dodecanoyl-2-hydroxy-*sn*-glycero-3-phosphocholine (NBD-lyso-PC), N-hexanoyl-NBD-sphingosine-1-phosphocholine (NBD-SM), N-hexanoyl-NBD-sphingosine (NBD-CER), N-hexanoyl-NBD-sphingosine β-D-galactosyl (NBD-LacCER), N-hexanoyl-NBD-sphingosine β-D-glucosyl (NBD-GluCer), NBD-sphingosine-1-phosphocholine (NBD-Lyso-SM), NBD-sphingosine-1-phosphate (NBD-SPH1P), and NBD-sphingosine (NBD-SPH) were purchased from Avanti Polar Lipids (Birmingham, AL, USA). All NBD-lipid stocks (4 mM, except NBD-SPH1P (10 mM)) were prepared in DMSO.

Uptake experiments in yeast were performed essentially as described previously[9]. Briefly, cells were resuspended to 10 $OD_{600}$/mL and incubated in selective SG medium followed by labeling with ~32 µM NBD-lipids for 30 min at 30°C with periodic mixing. Before analysis by flow cytometry, cells were washed three times in ice-cold selection media lacking galactose, but containing 2% (w/v) sorbitol, 3% (w/v) bovine serum albumin, and 20 mM $NaN_3$.

Lipid uptake assays in *Arabidopsis* were performed as described[9]. Five-day-old plate-grown *Arabidopsis* seedlings were incubated for the indicated times at room temperature in ½ Murashige and Skoog (MS) liquid medium in the presence of 40 µM NBD-SM. Periodic agitation was applied to facilitate diffusion of the lipid. After incubation, plants were washed twice with ½ MS media without lipid and visualized within two hours. For lipid analysis, plants were incubated as described in the presence of NBD-SM for 30 min. After washing, the plants were kept at room temperature without agitation in liquid ½ MS media for the indicated time periods, to allow for metabolic conversion of the fluorescent lipid.

***Flow cytometry***

Flow cytometry was performed on a Becton Dickinson FACS equipped with an argon laser using Cell Quest software. Prior to analysis, $10^7$ cells were labeled with one microliter of 1 mg/mL propidium iodide (PI) for staining of non-viable cells. Twenty thousand cells were analyzed. Data were analyzed using Cyflogic (CyFlo, Ltd). Viable yeast cells were selected based on forward/side-scatter gating and PI exclusion. NBD-fluorescence of living cells was plotted on a histogram and the geometric-mean fluorescence intensity was used for further statistical analysis.

***Microscopy visualisation and quantification of fluorescence***

A Leica SP2 UV MP or SP5 II spectral confocal laser scanning microscope (Leica Microsystems, Heidelberg, Germany) was used for fluorescence microscopy. For visualisation of plant tissues, a 63x/1.2 N.A. water immersion objective was used and all yeast imaging was done using a 100x/1.40 N.A. oil immersion objective. NBD was excited at 458 nm and emission signals were recorded between 495 and 550 nm for both yeast and root tips.

Quantification of NBD signals in plant roots was carried out using ImageJ (http://imagej.nih.gov/ij/). Regions of interest (ROI) enclosing the root tip right under the elongation zone were defined for each image and average pixel intensity was measured in each region.

***Lipid analysis***

Following NBD-lipid uptake assays in yeast, total cellular lipids were extracted twice from 100 µl of cell suspension in 2 volumes of chloroform/methanol 1/1 (vol/vol). For plants, 10-15 NBD-lipid labelled seedlings were ground, resuspended in 100 µl ½MS media and extracted twice as above. For preparation of root extracts, the roots from 10-15 five-day-old seedlings were cut and ground in the presence of ½ MS medium (pH 5.7), before addition of NBD-lipids and incubation as indicated for whole seedlings above. After incubation, samples were centrifuged shortly to remove debris and the supernatant was collected for total lipid extraction. In all cases, the lipid containing chloroform/methanol phase was dried and resuspended in a small volume of chloroform before separation by thin-layer chromatography (TLC) using chloroform/ethanol/water/triethylamine (30/35/7/35, vol/vol/vol/vol). NBD-lipid standards were chromatographed on the same plate. Fluorescent lipid spots were visualised with a Typhoon Trio Variable Mode Imager (GE Healthcare, Brøndby, Denmark). Original TLC scans can be found in Supplementary Datasets S2 and S3.

***Statistical analysis***

Data were analyzed using Excel (Microsoft). Data represent means ± s.e.m. of at least 3 experiments. Statistical analysis of sphingolipid transport data with respect to empty vector controls in Figure 2**a** was carried out using a Student's t-test, with a two-tailed distribution and two-sample unequal variance. For lipid uptake assays in planta, a single-factor analysis of variance (ANOVA) followed by Dunnett's test was performed independently for each time point. For lipid uptake assays in yeast (Figs.4-6), a background correction was made by subtracting the raw values of NBD-fluorescence obtained for empty vector controls from the corresponding values obtained for each *ala10* mutant and each lipid in the same experiment. Then, the corrected values for each mutant and lipid were normalized to the corresponding value for the wild type version of ALA10, which was set to 100%. Statistics on transport rates with respect to wild type proteins (Fig. 3**b** and 3**c** and Supplementary Figs. S2, S3 and S4) were performed using two-factor analysis of variance (ANOVA) with blocking followed by a Tukey's honest significant difference (HSD) test.

**References to Supplementary Methods**


1. Shinoda, T., Ogawa, H., Cornelius, F. & Toyoshima, C. Crystal structure of the sodium-potassium pump at 2.4 A resolution. *Nature* **459,** 446–50 (2009).

2. Toyoshima, C., Nomura, H. & Tsuda, T. Lumenal gating mechanism revealed in calcium pump crystal structures with phosphate analogues. *Nature* **432,** 361–8 (2004).

3. Wang, K. *et al.* Structure and mechanism of Zn2+-transporting P-type ATPases. *Nature* **514,** 518–522 (2014).

4. Bublitz, M., Poulsen, H., Morth, J. P. & Nissen, P. In and out of the cation pumps: P-Type ATPase structure revisited. *Curr. Opin. Struct. Biol.* **20,** 431–439 (2010).

5. Edgar, R. C. MUSCLE: multiple sequence alignment with high accuracy and high throughput. *Nucleic Acid Res.* **32,** 1792–1797 (2004).

6. Trott, O. & Olson, A. J. AutoDock Vina: Improving the speed and accuracy of docking with a new scoring function, efficient optimization, and multithreading. *J. Comput. Chem.* **31,** 455–461 (2010).

7. Dunbrack, R. L. Rotamer libraries in the 21st century. *Curr. Opin. Struct. Biol.* **12,** 431–440 (2002).

8. Pettersen, E. F. *et al.* UCSF Chimera - A visualization system for exploratory research and analysis. *J. Comput. Chem.* **25,** 1605–1612 (2004).

9. Poulsen, L. R. *et al.* A phospholipid uptake system in the model plant Arabidopsis thaliana. *Nat. Commun.* **6,** 7649 (2015).

10. Poulsen, L. R. *et al.* The Arabidopsis P4-ATPase ALA3 Localizes to the Golgi and Requires a beta-Subunit to Function in Lipid Translocation and Secretory Vesicle Formation. *Plant Cell* **20,** 658–676 (2008).

11. López-Marqués, R. L., Poulsen, L. R. & Palmgren, M. G. A putative plant aminophospholipid flippase,

the arabidopsis p4 atpase ala1, localizes to the plasma membrane following association with a β-subunit. *PLoS One* **7,** e33042 (2012).

12. Hua, Z., Fatheddin, P. & Graham, T. R. An Essential Subfamily of Drs2p-related P-Type ATPases Is Required for Protein Trafficking between Golgi Complex and Endosomal/Vacuolar System. *Mol. Biol. Cell* **13,** 3162–3177 (2002).

13. Costa, S. *et al.* Role of posttranslational modifications at the β-subunit ectodomain in complex association with a promiscuous plant P4-ATPase. *Biochem. J.* **473,** 1605–1615 (2016).

14. Daniel Gietz, R. & Woods, R. A. *Guide to Yeast Genetics and Molecular and Cell Biology - Part B. Methods in Enzymology* **350,** (Elsevier, 2002).

15. Liu, Y. *et al.* Accurate assessment of antibiotic susceptibility and screening resistant strains of a bacterial population by linear gradient plate. *Sci. China. Life Sci.* **54,** 953–60 (2011).

16. López-Marqués, R. L. *et al.* Intracellular targeting signals and lipid specificity determinants of the ALA/ALIS P4-ATPase complex reside in the catalytic ALA alpha-subunit. *Mol. Biol. Cell* **21,** 791–801 (2010).

## Supplementary Figures

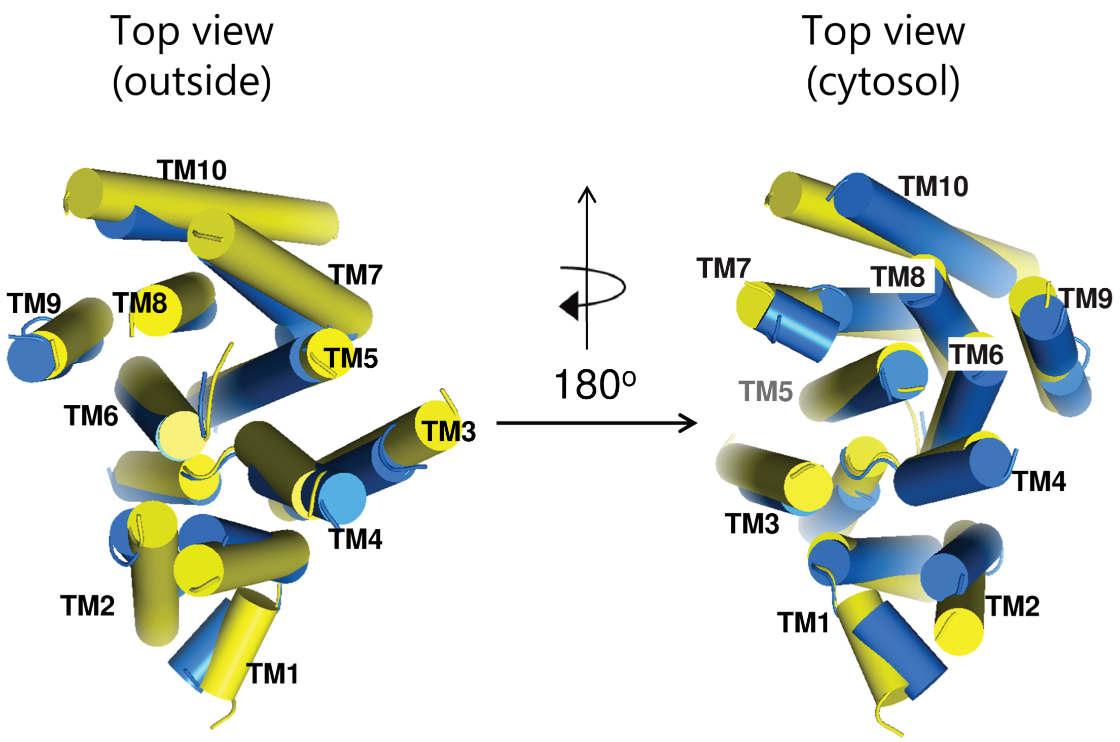


**Fig. S1: ALA10 homology model comparison to SERCA.** The transmembrane domains TM1-TM10 of the ALA10 homology model (blue) were aligned along TM4 to the SERCA structure used during generation of the model (pdb id. 3AR4, yellow).

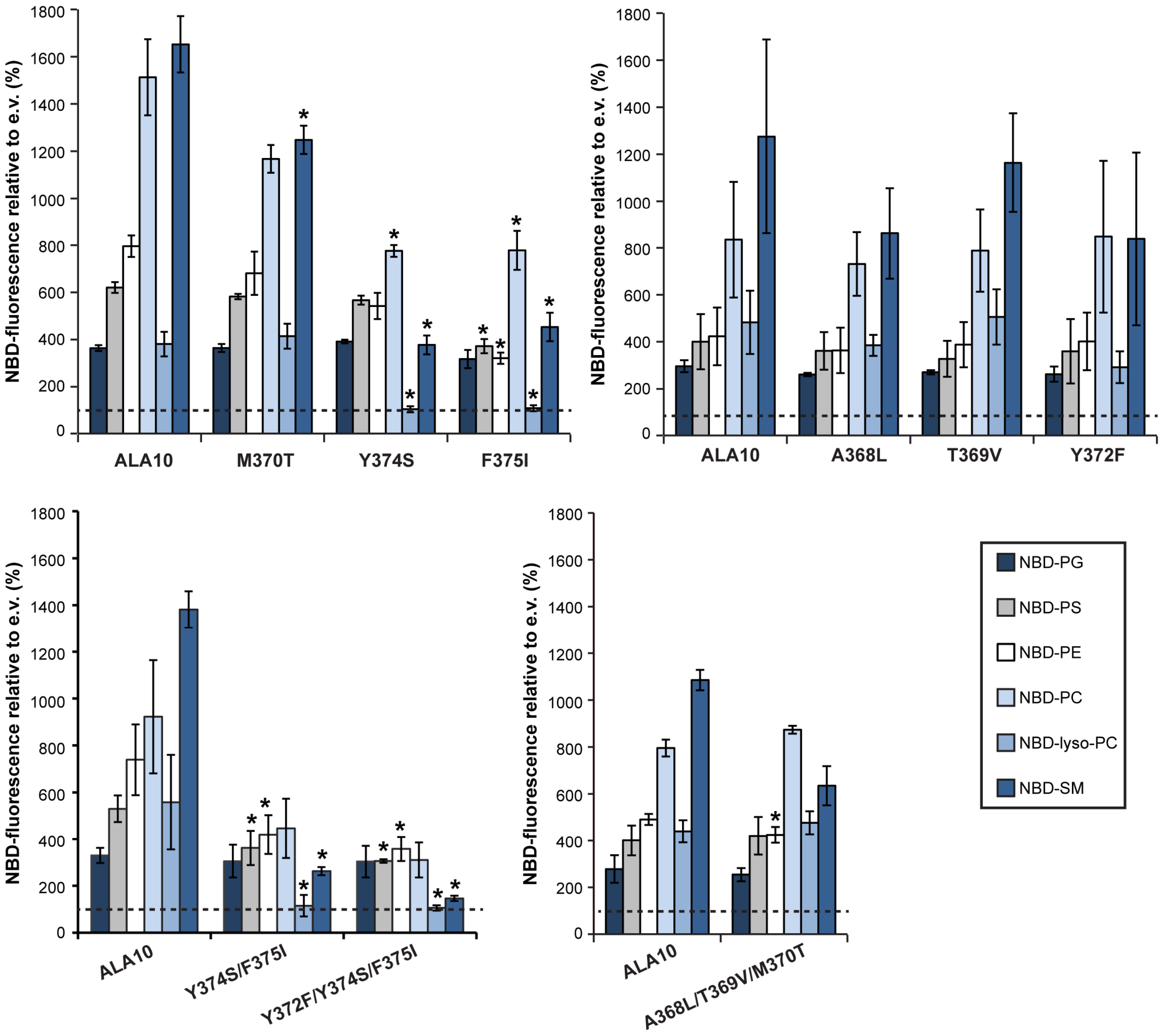


**Fig. S2. NBD-lipid transport by *ala10* TM4 point mutants.** Accumulation of NBD-lipids by *drs2Δdnf1,2Δ* yeast cells expressing the indicated *ala10* TM4 point mutants together with the β-subunit *ALIS5* (for details, see Materials and Methods). Values are normalized to *drs2Δdnf1,2Δ* cells transformed with empty vectors (set to 100%, dotted line). Results are averages ±s.e.m. of at least three independent experiments. Data were analysed using a two-factor ANOVA followed by Tukey's HSD test: *, $p<0.05$, significantly different with respect to wildtype ALA10.

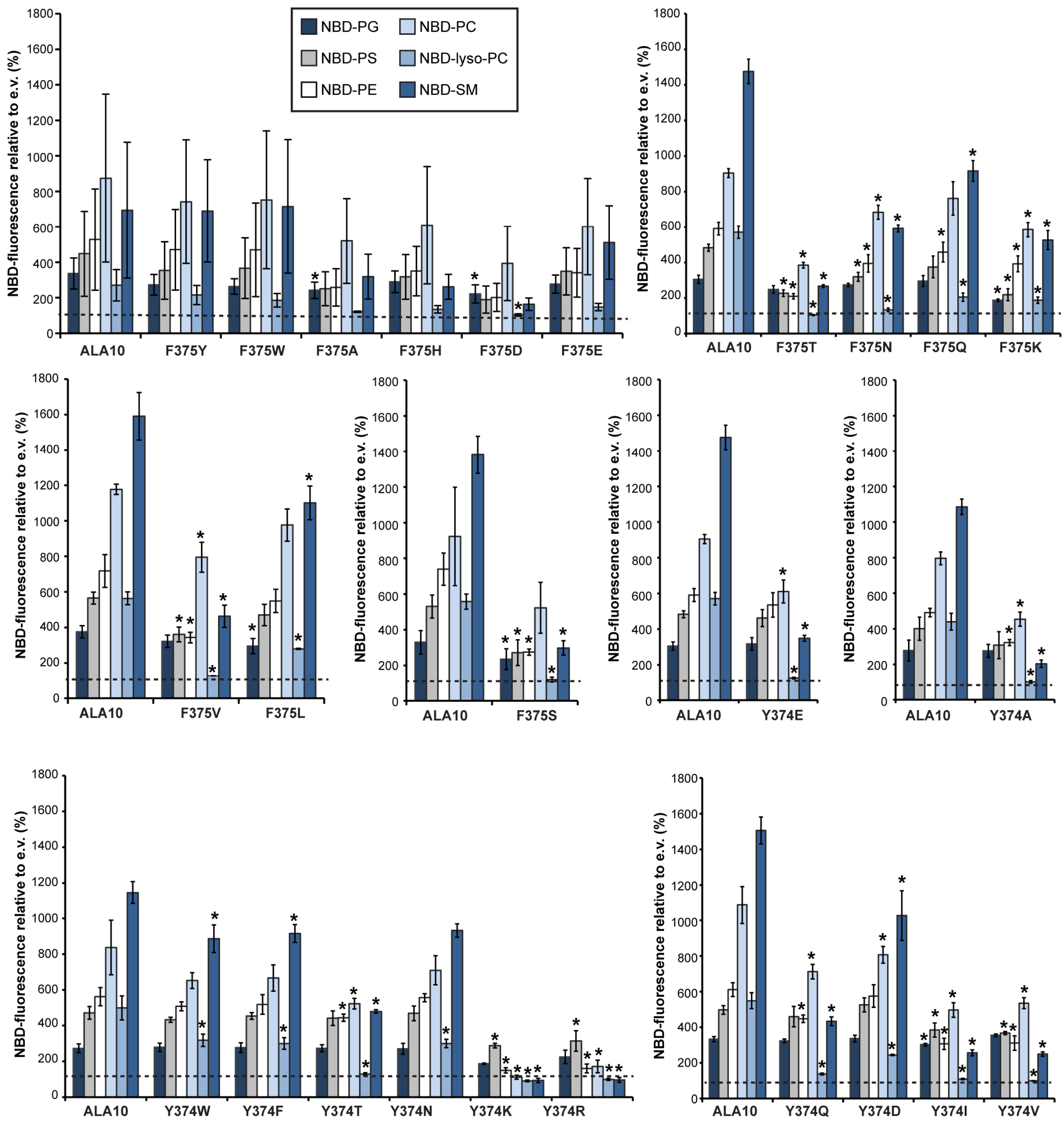


**Fig. S3. Changes in the side chain of the amino acid residues at positions 374 and 375 affect ALA10 lipid specificity.** Accumulation of NBD-lipids by *drs2Δdnf1,2Δ* cells transformed with *ala10* point mutants in Y374 or F375, as indicated, and ALIS5 is shown as relative fluorescence intensity with respect to *drs2Δdnf1,2Δ cells* transformed with empty vector (e.v.) controls. Results are averages ±s.e.m. of at least three independent experiments. Data were analysed using a two-factor ANOVA followed by Tukey's HSD test: *, $p<0.05$, significantly different with respect to wildtype ALA10.

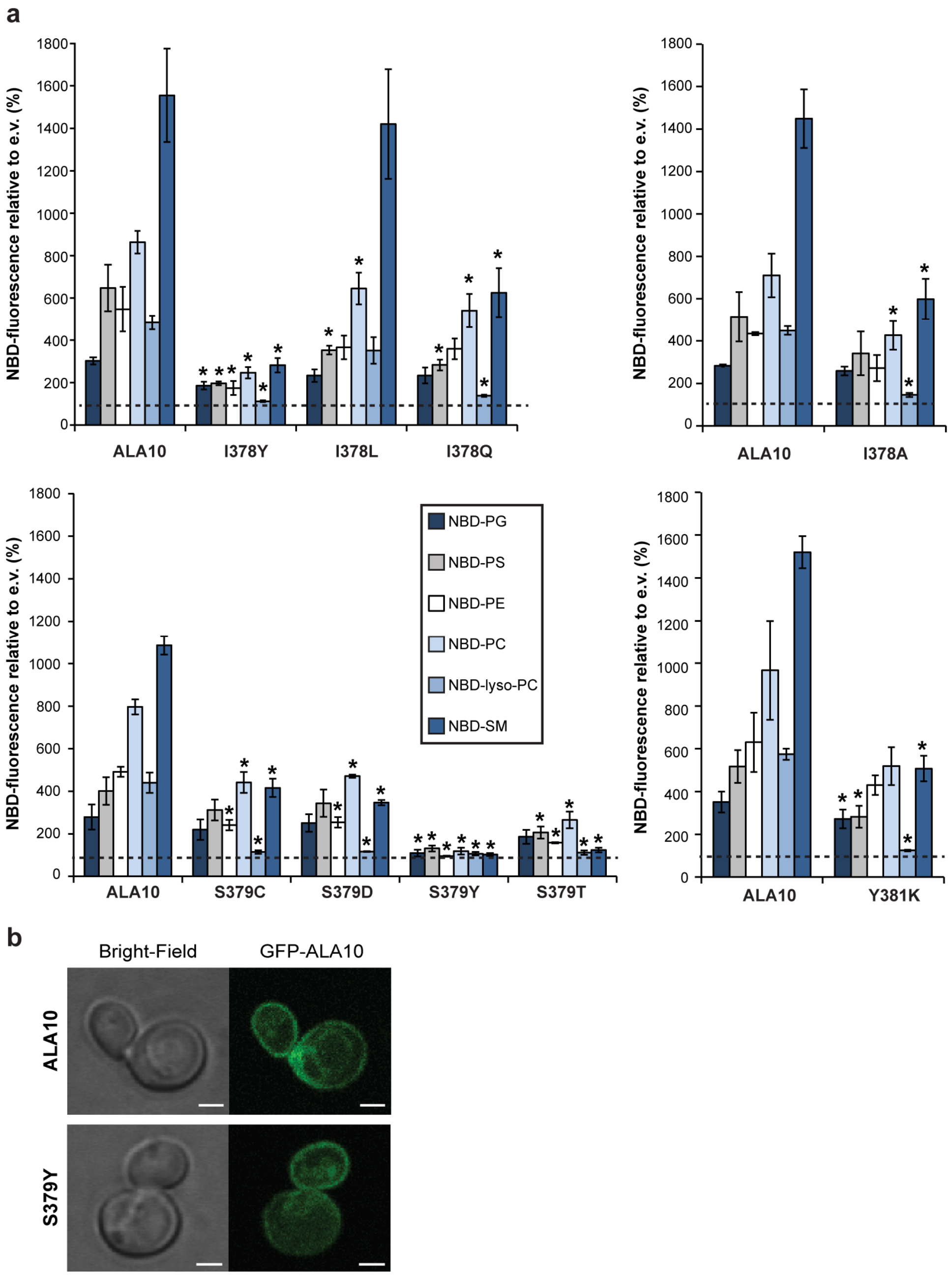


**Fig. S4. Changes in the side chain of the amino acid residues located after a conserved proline in TM4 affect ALA10 lipid specificity.** (**a**) Accumulation of NBD-lipids by *drs2Δdnf1,2Δ* cells transformed with *ala10* point mutants in I378, S379 or Y381, as indicated, and ALIS5 is shown as relative fluorescence intensity with respect to *drs2Δdnf1,2Δ cells* transformed with empty vector (e.v.) controls. Results are averages ± s.e.m. of at least three independent experiments. Data were analysed using a two-factor ANOVA followed by Tukey's HSD test: *, $p<0.05$, significantly different with respect to wild type ALA10. (**b**) Subcellular localization of GFP-ALA10 and GFP-ala10S379Y in the presence of ALIS1 upon expression in *drs2Δdnf1,2Δ* cells. Scale bar, 5 µm.

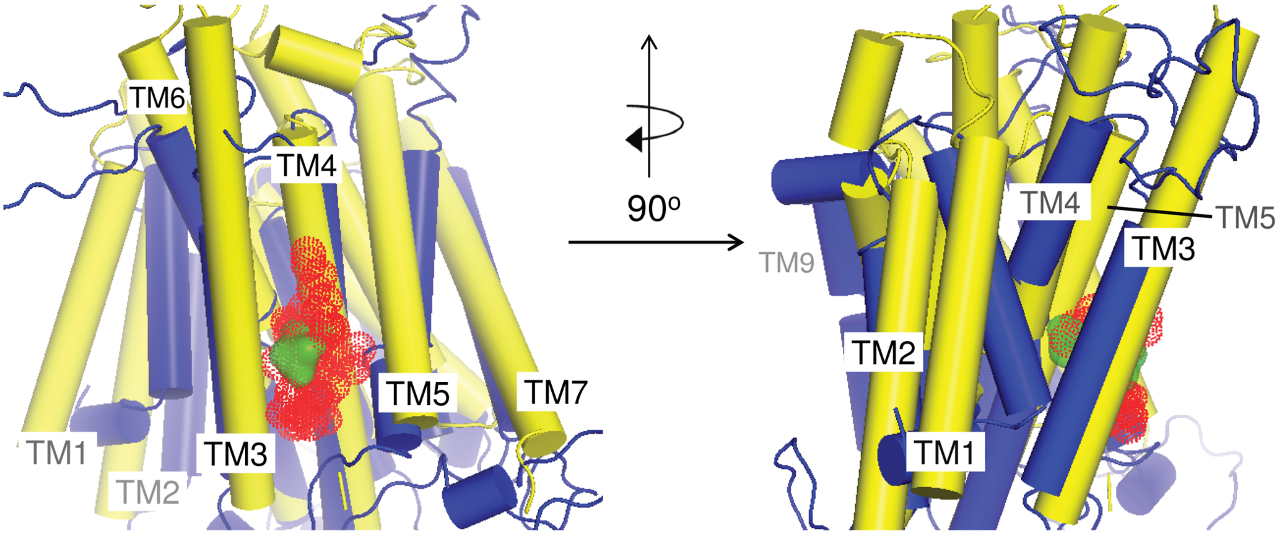


**Fig. S5. A hydrophobic central cavity formed between transmembrane domain TM3, TM4, and TM5 is present in cation-transporting P-type ATPases.** The crystal structure of the calcium SERCA pump with bound thapsigargin (3AR4, in yellow) was aligned along TM4 to the ALA10 structural model (in blue), using the PyMOL Molecular Graphics System, Version 1.8 (Schrödinger, LLC). Red dots represent the surface of bound thapsigargin, while a green surface indicates a phosphocholine headgroup.

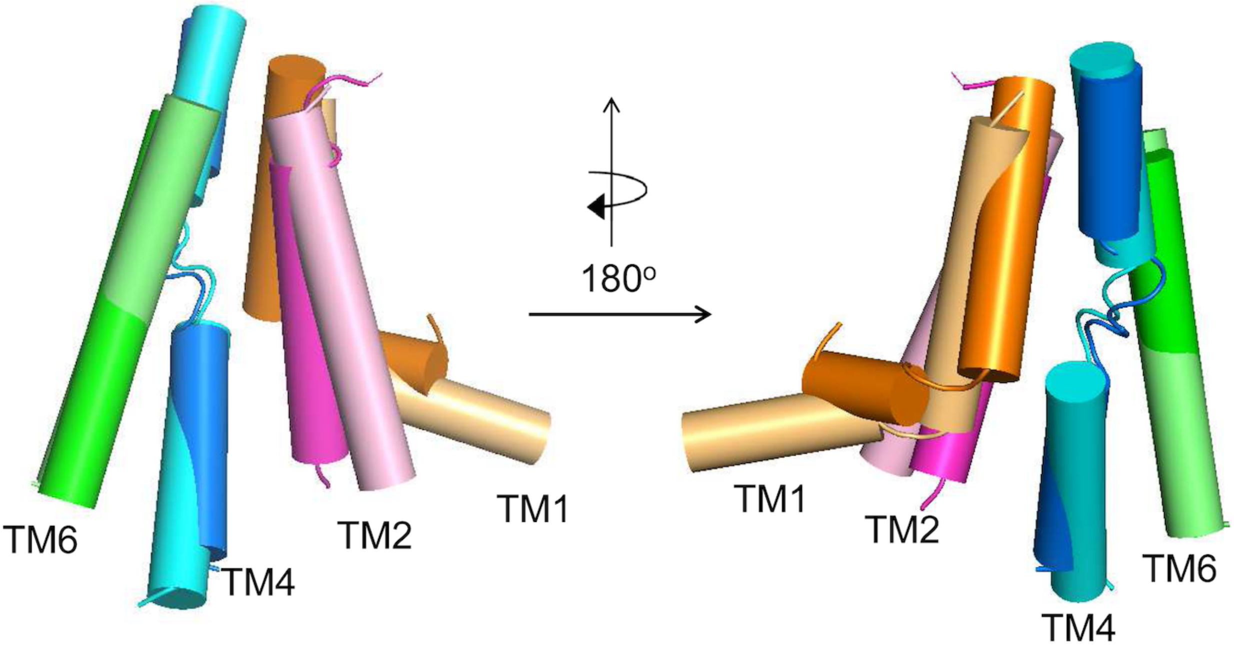


**Fig. S6. Relative positions of transmembrane domains TM1, TM2, TM4, and TM6 in the structural models of ATP8A2 and ALA10.** The ATP8A2 structural model in the E2P conformation (http://www.modelarchive.org/project/index/doi/ma-ax7dd, in pale colours) was aligned along TM4 and TM6 to the ALA10 structural model (in bright colors), using the PyMOL Molecular Graphics System, Version 1.8 (Schrödinger, LLC). Note that the separation of TM1 and TM2 from TM4 and TM6 is smaller in ALA10 than in ATP8A2, which would prevent a lipid from using this pathway to enter the protein.

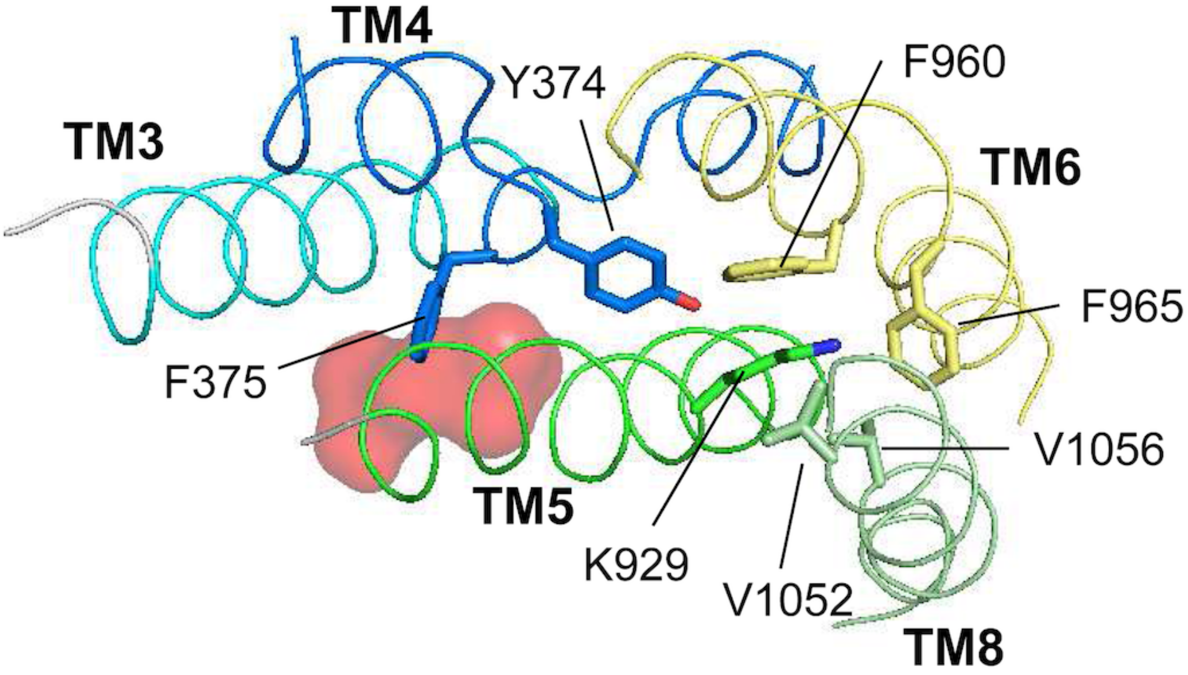


**Fig. S7. The conserved K929 residue in TM5 might form a hydrogen bond with Y374 in transmembrane domain TM4.** Outside view of the transmembrane region of the ALA10 structural model showing the position of a conserved lysine residue in TM5 essential for lipid translocation (K929). A red surface represents the phosphocholine headgroup embedded in the transmembrane central cavity. In the ALA10 structural model, the conserved lysine points away from the central cavity in a position that would allow it to form a hydrogen bond with the side chain of Y374 in TM4, thus involving this lysine in controlling the dynamics of the central cavity. Notably, residues in TM6 and TM8 located close to K929 are all aromatic or hydrophobic.

**Supplementary Tables**

**Table S1.** Templates and primers used in PCR amplifications for plasmid construction

| Template | Target construct | PCR | Primers |
|---|---|---|---|
| **pMP4133** | *ala3-ALA10TM4* | PCR 1 | **oli2796** 5'CGGCTTCTAATCCGTACTTC'3<br><br>**oli4478** 5'AACGTAAAGCGAAATCGGGATGAAGTAACTATACAGCATTGTAGCAGTGAAGAAGGTGAAAAATCCTATCATGAGTCCA'3 |
| | | PCR 2 | **oli4477** 5'TTCTTCACTGCTACAATGCTGTATA GTTACTTCATCCCGATTTCGCTTTACGTTTCTATTGAGATGATCAAGTTTATCC'3<br><br>**oli4351** 5'CTCTTCGCTATTACGCCAGC'3 |
| **pMP3136** | *ala10-ALA3TM4* | PCR 1 | **oli4312** 5'CTTTTGCTATTGTCATCAAGCTACGAAGAT'3<br><br>**oli4132** 5'GAAACATAAAGGGAGATTGGTATAATTGATGAGAACAGAGTGACTAGCGTGAAAAAGTGGTATATTGCAGCCATTGGAGCTCTTTCTGGATCAAAGAAGATATC'3 |
| | | PCR 2 | **oli4133** 5'CTTTTTCACGCTAGTCACTCTGTTCTCATCAATTATACCAATCTCCCTTTATGTTTCGATTGAGATCGTTAAAGTTCTTCAGAGCATTTTCATCAATAGAGAC'3<br><br>**oli4313** 5'CGATCCTCGCTCACGGAAGCCTTAGCTTCA'3 |
| **pMP3136** | *ala10M370T* | PCR 1 | **oli4312** 5'CTTTTGCTATTGTCATCAAGCTACGAAGAT'3<br><br>**oli4951** 5'GAAGTAACTATACAGAGTTGTAGCAGTGAA'3 |
| | | PCR 2 | **oli4950** 5'TTCACTGCTACAACTCTGTATAGTTACTTC'3<br><br>**oli4313** 5'CGATCCTCGCTCACGGAAGCCTTAGCTTCA'3 |
| **pMP3136** | *ala10Y374S* | PCR 1 | **oli4312** 5'CTTTTGCTATTGTCATCAAGCTACGAAGAT'3<br><br>**oli4953** 5'CGAAATCGGGATGAATGAACTATACAGCAT'3 |
| | | PCR 2 | **oli4952** 5'ATGCTGTATAGTTCATTCATCCCGATTTCG'3<br><br>**oli4313** 5'CGATCCTCGCTCACGGAAGCCTTAGCTTCA'3 |
| **pMP3136** | *ala10F375I* | PCR 1 | **oli4312** 5'CTTTTGCTATTGTCATCAAGCTACGAAGAT'3<br><br>**oli4955** 5'AGCGAAATCGGGATAATGTAACTATACAGC'3 |
| | | PCR 2 | **oli4954** 5'GCTGTATAGTTACATTATCCCGATTTCGCT'3<br><br>**oli431**3 5'CGATCCTCGCTCACGGAAGCCTTAGCTTCA'3 |
| **pMP3136** | *ala10T369V* | PCR 1 | **oli4312** 5'CTTTTGCTATTGTCATCAAGCTACGAAGAT'3 |

| | | | |
|---|---|---|---|
| | | | **oli5028**<br>5’AAGTAACTATACAGCATGACAGCAGTGAAGAAGTGGTATA’3 |
| | | PCR 2 | **oli5027**<br>5’TATACCACTTCTTCACTGCTGTCATGCTGTATAGTTACTT’3<br><br>**oli4313** 5’CGATCCTCGCTCACGGAAGCCTTAGCTTCA’3 |
| **pMP3136** | *ala10A368L* | PCR 1 | **oli4312** 5’CTTTTGCTATTGTCATCAAGCTACGAAGAT’3<br><br>**oli5030**<br>5’AACTATACAGCATTGTTAGAGTGAAGAAGTGGTATATTGC’3 |
| | | PCR 2 | **oli5029**<br>5’GCAATATACCACTTCTTCACTCTAACAATGCTGTATAGTT’3<br><br>**oli4313** 5’CGATCCTCGCTCACGGAAGCCTTAGCTTCA’3 |
| **pMP3136** | *ala10Y372F* | PCR 1 | **oli4312** 5’CTTTTGCTATTGTCATCAAGCTACGAAGAT’3<br><br>**oli5032**<br>5’AATCGGGATGAAGTAACTGAACAGCATTGTAGCAGTGAAG’3 |
| | | PCR 2 | **oli5031**<br>5’CTTCACTGCTACAATGCTGTTCAGTTACTTCATCCCGATT’3<br><br>**oli4313** 5’CGATCCTCGCTCACGGAAGCCTTAGCTTCA’3 |
| **pMP3136** | *ala10F375Y* | PCR 1 | **oli4312** 5’CTTTTGCTATTGTCATCAAGCTACGAAGAT’3<br><br>**oli5034** 5’GCGAAATCGGGATGTAGTAACTATACAG’3 |
| | | PCR 2 | **oli5033** 5’CTGTATAGTTACTACATCCCGATTTCGC’3<br><br>**oli4313** 5’CGATCCTCGCTCACGGAAGCCTTAGCTTCA’3 |
| **pMP3136** | *ala10F375W* | PCR 1 | **oli4312** 5’CTTTTGCTATTGTCATCAAGCTACGAAGAT’3<br><br>**oli5036** 5’GCGAAATCGGGATCCAGTAACTATACAG’3 |
| | | PCR 2 | **oli5035** 5’CTGTATAGTTACTGGATCCCGATTTCGC’3<br><br>**oli4313** 5’CGATCCTCGCTCACGGAAGCCTTAGCTTCA’3 |
| **pMP3136** | *ala10F375A* | PCR 1 | **oli4312** 5’CTTTTGCTATTGTCATCAAGCTACGAAGAT’3<br><br>**oli5038** 5’GCGAAATCGGGATGGCGTAACTATACAG’3 |
| | | PCR 2 | **oli5037** 5’CTGTATAGTTACGCCATCCCGATTTCGC’3<br><br>**oli4313** 5’CGATCCTCGCTCACGGAAGCCTTAGCTTCA’3 |
| **pMP3136** | *ala10F375H* | PCR 1 | **oli4312** 5’CTTTTGCTATTGTCATCAAGCTACGAAGAT’3<br><br>**oli5040** 5’GCGAAATCGGGATGTGGTAACTATACAG’3 |
| | | PCR 2 | **oli5039** 5’CTGTATAGTTACCACATCCCGATTTCGC’3<br><br>**oli4313** 5’CGATCCTCGCTCACGGAAGCCTTAGCTTCA’3 |

| | | | |
|---|---|---|---|
| **pMP3136** | *ala10F375D* | PCR 1 | **oli4312** 5’CTTTTGCTATTGTCATCAAGCTACGAAGAT’3<br>**oli5042** 5’GCGAAATCGGGATGTCGTAACTATACAG’3 |
| | | PCR 2 | **oli5041** 5’CTGTATAGTTACGACATCCCGATTTCGC’3<br>**oli4313** 5’CGATCCTCGCTCACGGAAGCCTTAGCTTCA’3 |
| **pMP3136** | *ala10F375E* | PCR 1 | **oli4312** 5’CTTTTGCTATTGTCATCAAGCTACGAAGAT’3<br>**oli5055** 5’GCGAAATCGGGATCTCGTAACTATACAG’3 |
| | | PCR 2 | **oli5054** 5’CTGTATAGTTACGAGATCCCGATTTCGC’3<br>**oli4313** 5’CGATCCTCGCTCACGGAAGCCTTAGCTTCA’3 |
| **pMP3136** | *ala10F375V* | PCR 1 | **oli4312** 5’CTTTTGCTATTGTCATCAAGCTACGAAGAT’3<br>**oli5047** 5’GCGAAATCGGGATGACGTAACTATACAG’3 |
| | | PCR 2 | **oli5046** 5’CTGTATAGTTACGTCATCCCGATTTCGC’3<br>**oli4313** 5’CGATCCTCGCTCACGGAAGCCTTAGCTTCA’3 |
| **pMP3136** | *ala10F375L* | PCR 1 | **oli4312** 5’CTTTTGCTATTGTCATCAAGCTACGAAGAT’3<br>**oli5049** 5’GCGAAATCGGGATGAGGTAACTATACAG’3 |
| | | PCR 2 | **oli5048** 5’CTGTATAGTTACCTCATCCCGATTTCGC’3<br>**oli4313** 5’CGATCCTCGCTCACGGAAGCCTTAGCTTCA’3 |
| **pMP3136** | *ala10F375S* | PCR 1 | **oli4312** 5’CTTTTGCTATTGTCATCAAGCTACGAAGAT’3<br>**oli5051** 5’GCGAAATCGGGATGCTGTAACTATACAG’3 |
| | | PCR 2 | **oli5050** 5’CTGTATAGTTACAGCATCCCGATTTCGC’3<br>**oli4313** 5’CGATCCTCGCTCACGGAAGCCTTAGCTTCA’3 |
| **pMP3136** | *ala10F375T* | PCR 1 | **oli4312** 5’CTTTTGCTATTGTCATCAAGCTACGAAGAT’3<br>**oli5053** 5’GCGAAATCGGGATGGTGTAACTATACAG’3 |
| | | PCR 2 | **oli5052** 5’CTGTATAGTTACACCATCCCGATTTCGC’3<br>**oli4313** 5’CGATCCTCGCTCACGGAAGCCTTAGCTTCA’3 |
| **pMP3136** | *ala10F375N* | PCR 1 | **oli4312** 5’CTTTTGCTATTGTCATCAAGCTACGAAGAT’3<br>**oli5057** 5’GCGAAATCGGGATGTTGTAACTATACAG’3 |
| | | PCR 2 | **oli5056** 5’CTGTATAGTTACAACATCCCGATTTCGC’3<br>**oli4313** 5’CGATCCTCGCTCACGGAAGCCTTAGCTTCA’3 |
| **pMP3136** | *ala10F375Q* | PCR 1 | **oli4312** 5’CTTTTGCTATTGTCATCAAGCTACGAAGAT’3<br>**oli5059** 5’GCGAAATCGGGATCTGGTAACTATACAG’3 |
| | | PCR 2 | **oli5058** 5’CTGTATAGTTACCAGATCCCGATTTCGC’3 |

| | | | |
|---|---|---|---|
| | | | **oli4313** 5’CGATCCTCGCTCACGGAAGCCTTAGCTTCA’3 |
| **pMP3136** | *ala10F375K* | PCR 1 | **oli4312** 5’CTTTTGCTATTGTCATCAAGCTACGAAGAT’3<br><br>**oli5241** 5’GCGAAATCGGGATCTTGTAACTATACAG’3 |
| | | PCR 2 | **oli5240** 5’CTGTATAGTTACAAGATCCCGATTTCGC’3<br><br>**oli4313** 5’CGATCCTCGCTCACGGAAGCCTTAGCTTCA’3 |
| **pMP3136** | *ala10Y374E* | PCR 1 | **oli4312** 5’CTTTTGCTATTGTCATCAAGCTACGAAGAT’3<br><br>**oli5061** 5’CGAAATCGGGATGAACTCACTATACAGC’3 |
| | | PCR 2 | **oli5060** 5’GCTGTATAGTGAGTTCATCCCGATTTCG’3<br><br>**oli4313** 5’CGATCCTCGCTCACGGAAGCCTTAGCTTCA’3 |
| **pMP3136** | *ala10Y374A* | PCR 1 | **oli4312** 5’CTTTTGCTATTGTCATCAAGCTACGAAGAT’3<br><br>**oli5073** 5’CGAAATCGGGATGAAGGCACTATACAGC’3 |
| | | PCR 2 | **oli5072** 5’GCTGTATAGTGCCTTCATCCCGATTTCG’3<br><br>**oli4313** 5’CGATCCTCGCTCACGGAAGCCTTAGCTTCA’3 |
| **pMP3136** | *ala10Y374W* | PCR 1 | **oli4312** 5’CTTTTGCTATTGTCATCAAGCTACGAAGAT’3<br><br>**oli5075** 5’CGAAATCGGGATGAACCAACTATACAGC’3 |
| | | PCR 2 | **oli5074** 5’GCTGTATAGTTGGTTCATCCCGATTTCG’3<br><br>**oli4313** 5’CGATCCTCGCTCACGGAAGCCTTAGCTTCA’3 |
| **pMP3136** | *ala10Y374F* | PCR 1 | **oli4312** 5’CTTTTGCTATTGTCATCAAGCTACGAAGAT’3<br><br>**oli5077** 5’CGAAATCGGGATGAAGAAACTATACAGC’3 |
| | | PCR 2 | **oli5076** 5’GCTGTATAGTTTCTTCATCCCGATTTCG’3<br><br>**oli4313** 5’CGATCCTCGCTCACGGAAGCCTTAGCTTCA’3 |
| **pMP3136** | *ala10Y374T* | PCR 1 | **oli4312** 5’CTTTTGCTATTGTCATCAAGCTACGAAGAT’3<br><br>**oli5079** 5’CGAAATCGGGATGAAGGTACTATACAGC’3 |
| | | PCR 2 | **oli5078** 5’GCTGTATAGTACCTTCATCCCGATTTCG’3<br><br>**oli4313** 5’CGATCCTCGCTCACGGAAGCCTTAGCTTCA’3 |
| **pMP3136** | *ala10Y374Q* | PCR 1 | **oli4312** 5’CTTTTGCTATTGTCATCAAGCTACGAAGAT’3<br><br>**oli5063** 5’CGAAATCGGGATGAACTGACTATACAGC’3 |
| | | PCR 2 | **oli5062** 5’GCTGTATAGTCAGTTCATCCCGATTTCG’3<br><br>**oli4313** 5’CGATCCTCGCTCACGGAAGCCTTAGCTTCA’3 |
| **pMP3136** | *ala10Y374D* | PCR 1 | **oli4312** 5’CTTTTGCTATTGTCATCAAGCTACGAAGAT’3<br><br>**oli5065** 5’CGAAATCGGGATGAAGTCACTATACAGC’3 |
| | | PCR 2 | **oli5064** 5’GCTGTATAGTGACTTCATCCCGATTTCG’3 |

| | | | |
|---|---|---|---|
| | | | **oli4313** 5’CGATCCTCGCTCACGGAAGCCTTAGCTTCA’3 |
| **pMP3136** | *ala10Y374I* | PCR 1 | **oli4312** 5’CTTTTGCTATTGTCATCAAGCTACGAAGAT’3<br><br>**oli5071** 5’CGAAATCGGGATGAAGACACTATACAGC’3 |
| | | PCR 2 | **oli5070** 5’GCTGTATAGTGTCTTCATCCCGATTTCG’3<br><br>**oli4313** 5’CGATCCTCGCTCACGGAAGCCTTAGCTTCA’3 |
| **pMP3136** | *ala10Y374V* | PCR 1 | **oli4312** 5’CTTTTGCTATTGTCATCAAGCTACGAAGAT’3<br><br>**oli5055** 5’GCGAAATCGGGATCTCGTAACTATACAG’3 |
| | | PCR 2 | **oli5054** 5’CTGTATAGTTACGAGATCCCGATTTCGC’3<br><br>**oli4313** 5’CGATCCTCGCTCACGGAAGCCTTAGCTTCA’3 |
| **pMP3136** | *ala10I378Y* | PCR 1 | **oli4313** 5’CGATCCTCGCTCACGGAAGCCTTAGCTTCA’3<br><br>**oli5544** 5’GTTACTTCATCCCGTATTCGCTTTACGTTTCGATTG’3 |
| | | PCR 2 | **oli4312** 5’CTTTTGCTATTGTCATCAAGCTACGAAGAT’3<br><br>**oli5545** 5’CAATCGAAACGTAAAGCGAATACGGGATGAAGTAAC’3 |
| **pMP3136** | *ala10I378L* | PCR 1 | **oli4313** 5’CGATCCTCGCTCACGGAAGCCTTAGCTTCA’3<br><br>**oli5548** 5’GTTACTTCATCCCGCTTTCGCTTTACGTTTCGATTG’3 |
| | | PCR 2 | **oli4312** 5’CTTTTGCTATTGTCATCAAGCTACGAAGAT’3<br><br>**oli5549** 5’CAATCGAAACGTAAAGCGAAAGCGGGATGAAGTAAC’3 |
| **pMP3136** | *ala10I378Q* | PCR 1 | **oli4313** 5’CGATCCTCGCTCACGGAAGCCTTAGCTTCA’3<br><br>**oli5558** 5’GTTACTTCATCCCGCAATCGCTTTACGTTTCGATTG’3 |
| | | PCR 2 | **oli4312** 5’CTTTTGCTATTGTCATCAAGCTACGAAGAT’3<br><br>**oli5559** 5’CAATCGAAACGTAAAGCGATTGCGGGATGAAGTAAC’3 |
| **pMP3136** | *ala10I378A* | PCR 1 | **oli4313** 5’CGATCCTCGCTCACGGAAGCCTTAGCTTCA’3<br><br>**oli5552** 5’GTTACTTCATCCCGGCTTCGCTTTACGTTTCGATTG’3 |
| | | PCR 2 | **oli4312** 5’CTTTTGCTATTGTCATCAAGCTACGAAGAT’3<br><br>**oli5553** 5’CAATCGAAACGTAAAGCGAAGCCGGGATGAAGTAAC’3 |
| **pMP3136** | *ala10S379C* | PCR 1 | **oli4313** 5’CGATCCTCGCTCACGGAAGCCTTAGCTTCA’3<br><br>**oli5082** 5’CTTCATCCCGATTTGCCTTTACGTTTC’3 |
| | | PCR 2 | **oli4312** 5’CTTTTGCTATTGTCATCAAGCTACGAAGAT’3<br><br>**oli5083** 5’GAAACGTAAAGGCAAATCGGGATGAAG’3 |
| **pMP3136** | *ala10S379D* | PCR 1 | **oli4313** 5’CGATCCTCGCTCACGGAAGCCTTAGCTTCA’3<br><br>**oli5084** 5’CTTCATCCCGATTGACCTTTACGTTTC’3 |
| | | PCR 2 | **oli4312** 5’CTTTTGCTATTGTCATCAAGCTACGAAGAT’3 |

| | | | |
|---|---|---|---|
| | | | **oli5085** 5'GAAACGTAAAGGTCAATCGGGATGAAG'3 |
| **pMP3136** | *ala10S379Y* | PCR 1 | **oli4313** 5'CGATCCTCGCTCACGGAAGCCTTAGCTTCA'3<br><br>**oli5086** 5'CTTCATCCCGATTTACCTTTACGTTTC'3 |
| | | PCR 2 | **oli4312** 5'CTTTTGCTATTGTCATCAAGCTACGAAGAT'3<br><br>**oli5087** 5'GAAACGTAAAGGTAAATCGGGATGAAG'3 |
| **pMP3136** | *ala10S379T* | PCR 1 | **oli4313** 5'CGATCCTCGCTCACGGAAGCCTTAGCTTCA'3<br><br>**oli5088** 5'CTTCATCCCGATTACGCTTTACGTTTC'3 |
| | | PCR 2 | **oli4312** 5'CTTTTGCTATTGTCATCAAGCTACGAAGAT'3<br><br>**oli5089** 5'GAAACGTAAAGCGTAATCGGGATGAAG'3 |
| **pMP3136** | *ala10Y381K* | PCR 1 | **oli4313** 5'CGATCCTCGCTCACGGAAGCCTTAGCTTCA'3<br><br>**oli4366** 5'GATCTCAATCGAAACCTTAAGCGAAATCGG'3 |
| | | PCR 2 | **oli4312** 5'CTTTTGCTATTGTCATCAAGCTACGAAGAT'3<br><br>**oli4367** 5'CCGATTTCGCTTAAGGTTTCGATTGAGATC'3 |
| **pMP3136** | *ala10Y374S F375I* | PCR 1 | **oli4313** 5'CGATCCTCGCTCACGGAAGCCTTAGCTTCA'3<br><br>**oli5026**<br>5'AAGCGAAATCGGGATAATTGAACTATACAGCATTGTAGCA'3 |
| | | PCR 2 | **oli4312** 5'CTTTTGCTATTGTCATCAAGCTACGAAGAT'3<br><br>**oli5025**<br>5'TGCTACAATGCTGTATAGTTCAATTATCCCGATTTCGCTT'3 |
| **pMP3136** | *ala10Y372F Y374SF375I* | PCR 1 | **oli4313** 5'CGATCCTCGCTCACGGAAGCCTTAGCTTCA'3<br><br>**oli5245** 5'GAAATCGGGATGATGCTACTAAACAGCATTGTAG'3 |
| | | PCR 2 | **oli4312** 5'CTTTTGCTATTGTCATCAAGCTACGAAGAT'3<br><br>**oli5244** 5'CTACAATGCTGTTTAGTAGCATCATCCCGATTTC'3 |
| **pMP3136** | *ala10A368L T369VM370T* | PCR 1 | **oli4313** 5'CGATCCTCGCTCACGGAAGCCTTAGCTTCA'3<br><br>**oli5304** 5'GAAGTAACTATACAGCGTTACAAGAGTGAAGAAGTGG'3 |
| | | PCR 2 | **oli4312** 5'CTTTTGCTATTGTCATCAAGCTACGAAGAT'3<br><br>**oli5303** 5'CCACTTCTTCACTCTTGTAACGCTGTATAGTTACTTC'3 |

**Table S2.** Plasmids used in this work

| Plasmid name | Plasmid backbone | Insert | Reference |
|---|---|---|---|
| **pMP1870** | pRS426-GAL | Empty vector | (1) |
| **pMP1872** | pRS423-GAL | Empty vector | (1) |
| **pMP2379** | pRS423-GAL | $RGSH_6$:*ALIS1* | (2) |
| **pMP3136** | pCR®4 Blunt-TOPO® | *ALA10* | (2) |
| **pMP3390** | pMP3157 | $RGSH_{10}$:*ALA10* | (3) |
| **pMP4133** | pRS423-GAL | $RGSH_{10}$:*ALA3* | This study |
| **pMP2382** | pRS426-GAL | $RGSH_6$:*ALIS5* | (2) |
| **pMP3912** | pMP3157 | $RGSH_{10}$:*ala10D430N* | (3) |
| **pMP4378** | pMP3157 | $RGSH_{10}$:*ala10-ALA3TM4* | This study |
| **pMP4750** | pMP3157 | $RGSH_{10}$:*ala10Y374SF375I* | This study |
| **pMP4751** | pMP3157 | $RGSH_{10}$:*ala10M370T* | This study |
| **pMP4752** | pMP3157 | $RGSH_{10}$:*ala10F375I* | This study |
| **pMP4753** | pMP3157 | $RGSH_{10}$:*ala10Y374S* | This study |
| **pMP4754** | pMP3157 | $RGSH_{10}$:*ala10T369V* | This study |
| **pMP4755** | pMP3157 | $RGSH_{10}$:*ala10A368L* | This study |
| **pMP4756** | pMP3157 | $RGSH_{10}$:*ala10Y372F* | This study |
| **pMP4757** | pMP3157 | $RGSH_{10}$:*ala10Y381K* | This study |
| **pMP4758** | pMP3157 | $RGSH_{10}$:*ala10F375Y* | This study |
| **pMP4759** | pMP3157 | $RGSH_{10}$:*ala10F375W* | This study |
| **pMP4760** | pMP3157 | $RGSH_{10}$:*ala10F375A* | This study |
| **pMP4761** | pMP3157 | $RGSH_{10}$:*ala10F375H* | This study |

| | | | |
|---|---|---|---|
| **pMP4762** | pMP3157 | *$RGSH_{10}$:ala10F375D* | This study |
| **pMP4763** | pMP3157 | *$RGSH_{10}$:ala10F375V* | This study |
| **pMP4764** | pMP3157 | *$RGSH_{10}$:ala10F375L* | This study |
| **pMP4765** | pMP3157 | *$RGSH_{10}$:ala10F375S* | This study |
| **pMP4766** | pMP3157 | *$RGSH_{10}$:ala10F375T* | This study |
| **pMP4767** | pMP3157 | *$RGSH_{10}$:ala10F375E* | This study |
| **pMP4768** | pMP3157 | *$RGSH_{10}$:ala10F375N* | This study |
| **pMP4769** | pMP3157 | *$RGSH_{10}$:ala10F375Q* | This study |
| **pMP4770** | pMP3157 | *$RGSH_{10}$:ala10Y372E* | This study |
| **pMP4771** | pMP3157 | *$RGSH_{10}$:ala10Y372Q* | This study |
| **pMP4772** | pMP3157 | *$RGSH_{10}$:ala10Y372D* | This study |
| **pMP4773** | pMP3157 | *$RGSH_{10}$:ala10Y372I* | This study |
| **pMP4775** | pMP3157 | *$RGSH_{10}$:ala10Y372V* | This study |
| **pMP4776** | pMP3157 | *$RGSH_{10}$:ala10Y372A* | This study |
| **pMP4777** | pMP3157 | *$RGSH_{10}$:ala10Y372W* | This study |
| **pMP4778** | pMP3157 | *$RGSH_{10}$:ala10Y372F* | This study |
| **pMP4779** | pMP3157 | *$RGSH_{10}$:ala10Y372T* | This study |
| **pMP4780** | pMP3157 | *$RGSH_{10}$:ala10Y372N* | This study |
| **pMP4781** | pMP3157 | *$RGSH_{10}$:ala10S379C* | This study |
| **pMP4782** | pMP3157 | *$RGSH_{10}$:ala10S379D* | This study |
| **pMP4783** | pMP3157 | *$RGSH_{10}$:ala10S379Y* | This study |
| **pMP4784** | pMP3157 | *$RGSH_{10}$:ala10S379T* | This study |
| **pMP4819** | pMP3157 | *$RGSH_{10}$:ala10Y372FY374SF375I* | This study |
| **pMP4820** | pMP3157 | *$RGSH_{10}$:ala10F375K* | This study |
| **pMP4822** | pMP3157 | *$RGSH_{10}$:ala10Y372K* | This study |
| **pMP4823** | pMP3157 | *$RGSH_{10}$:ala10Y372R* | This study |

| | | | |
|---|---|---|---|
| **pMP4883** | pMP4133 | *RGSH*$_{10}$*:ala3-ALA10TM4* | This study |
| **pMP4948** | pMP3157 | *RGSH*$_{10}$*:ala10A368LT369M370T* | This study |
| **pMP5192** | pMP3157 | *RGSH*$_{10}$*:ala10I378Y* | This study |
| **pMP5193** | pMP3157 | *RGSH*$_{10}$*:ala10I378L* | This study |
| **pMP5194** | pMP3157 | *RGSH*$_{10}$*:ala10I378Q* | This study |
| **pMP5195** | pMP3157 | *RGSH*$_{10}$*:ala10I378A* | This study |

**References to Supplementary Tables**


1. Burgers PMJ (1999) Overexpression of Multisubunit Replication Factors in Yeast. *Methods* 18(3):349–355.

2. Poulsen LR, et al. (2008) The Arabidopsis P4-ATPase ALA3 Localizes to the Golgi and Requires a beta-Subunit to Function in Lipid Translocation and Secretory Vesicle Formation. *Plant Cell* 20(3):658–676.

3. Poulsen LR, et al. (2015) A phospholipid uptake system in the model plant Arabidopsis thaliana. *Nat Commun* 6:7649.

**Supplementary Dataset S1:** Sequence alignment used to generate the ALA10 homology model. For details see the Supplementary Methods.

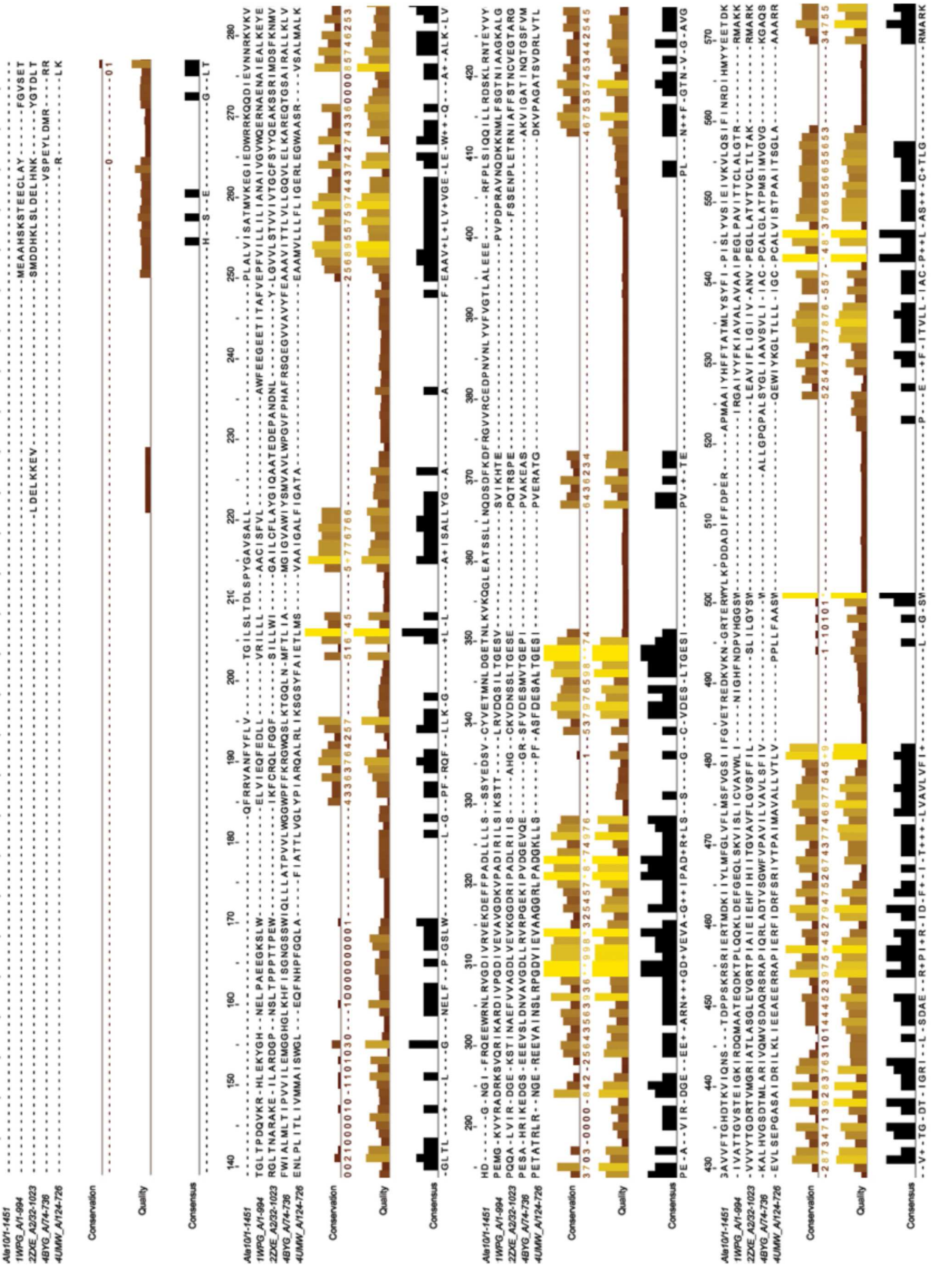

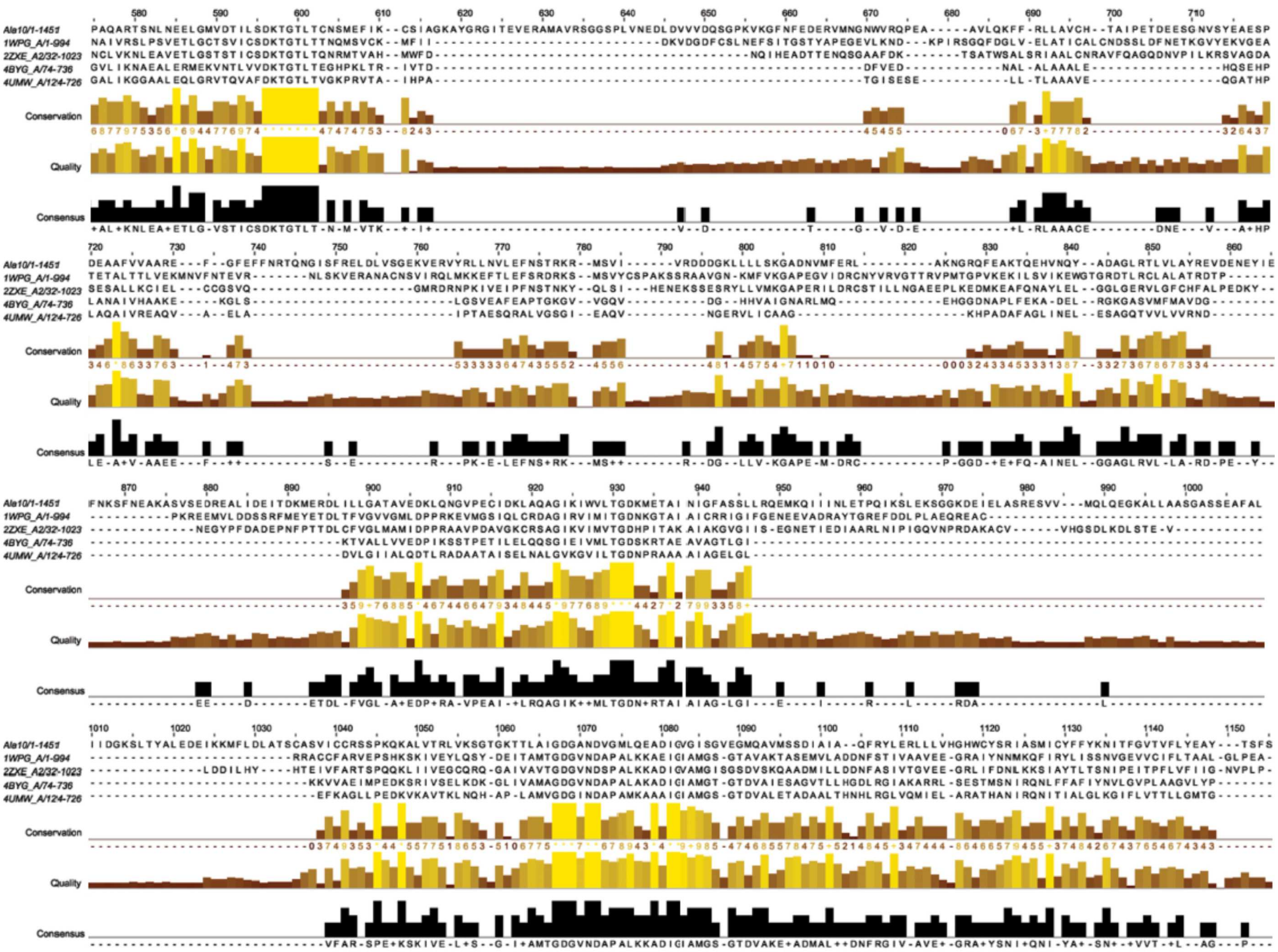
Ala10/1-1451
1WPG_A/1-994
2ZXE_A2/32-1023
4BYG_A/74-736
4UMW_A/124-726
Conservation
Quality
Consensus

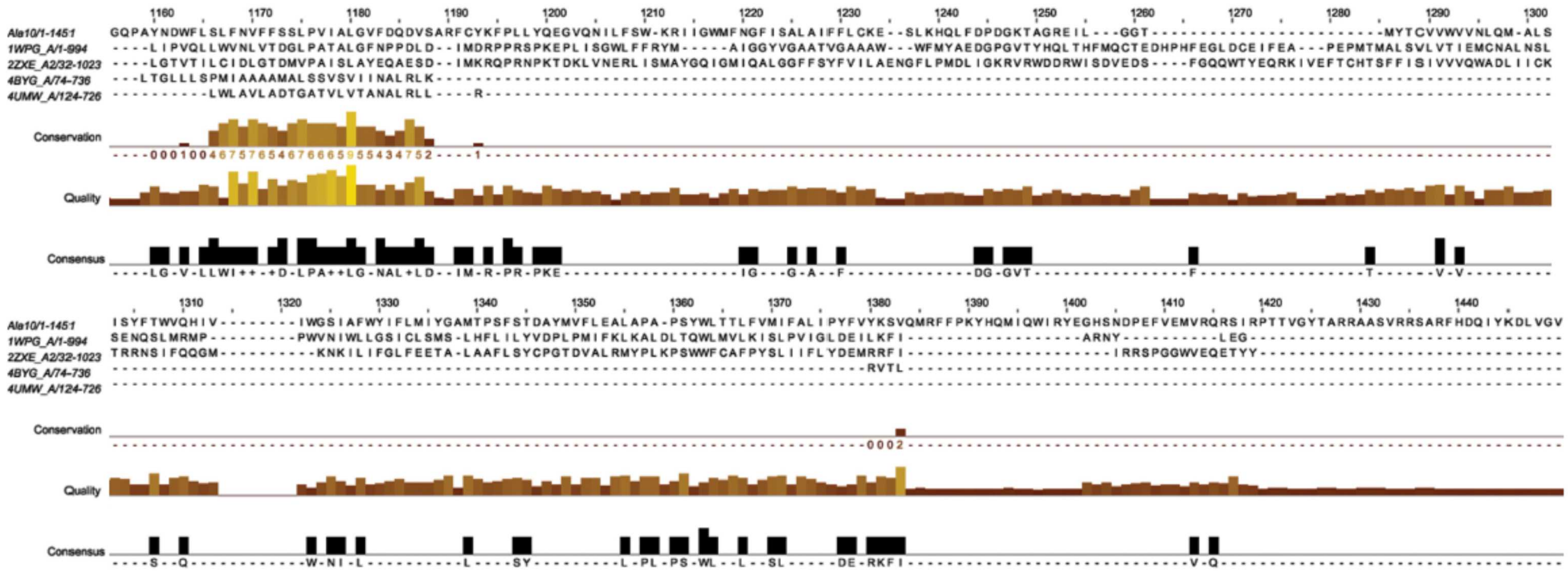

1160 1170 1180 1190 1200 1210 1220 1230 1240 1250 1260 1270 1280 1290 1300
Ala10/1-1451
1WPG_A/1-994
2ZXE_A2/32-1023
4BYG_A/74-736
4UMW_A/124-726
Conservation
Quality
Consensus
1310 1320 1330 1340 1350 1360 1370 1380 1390 1400 1410 1420 1430 1440
Ala10/1-1451
1WPG_A/1-994
2ZXE_A2/32-1023
4BYG_A/74-736
4UMW_A/124-726
Conservation
Quality
Consensus

**Supplementary Dataset S2**: Original scans for the TLC plates used in Figure 2 at different exposure times.

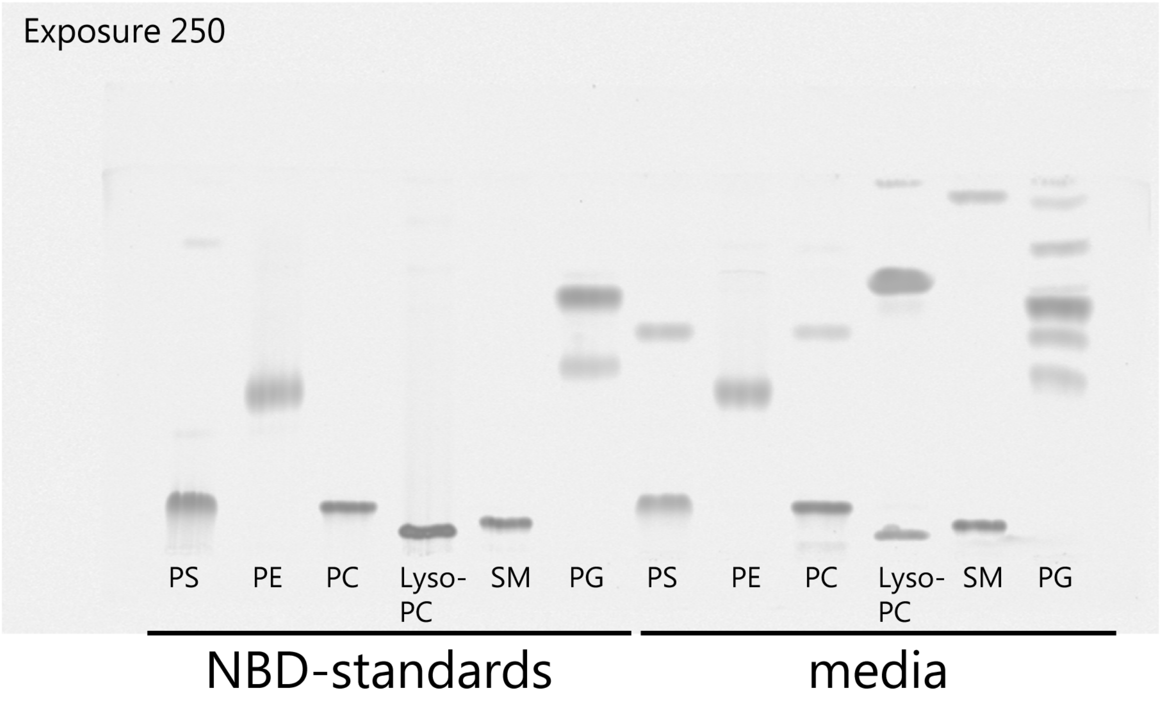


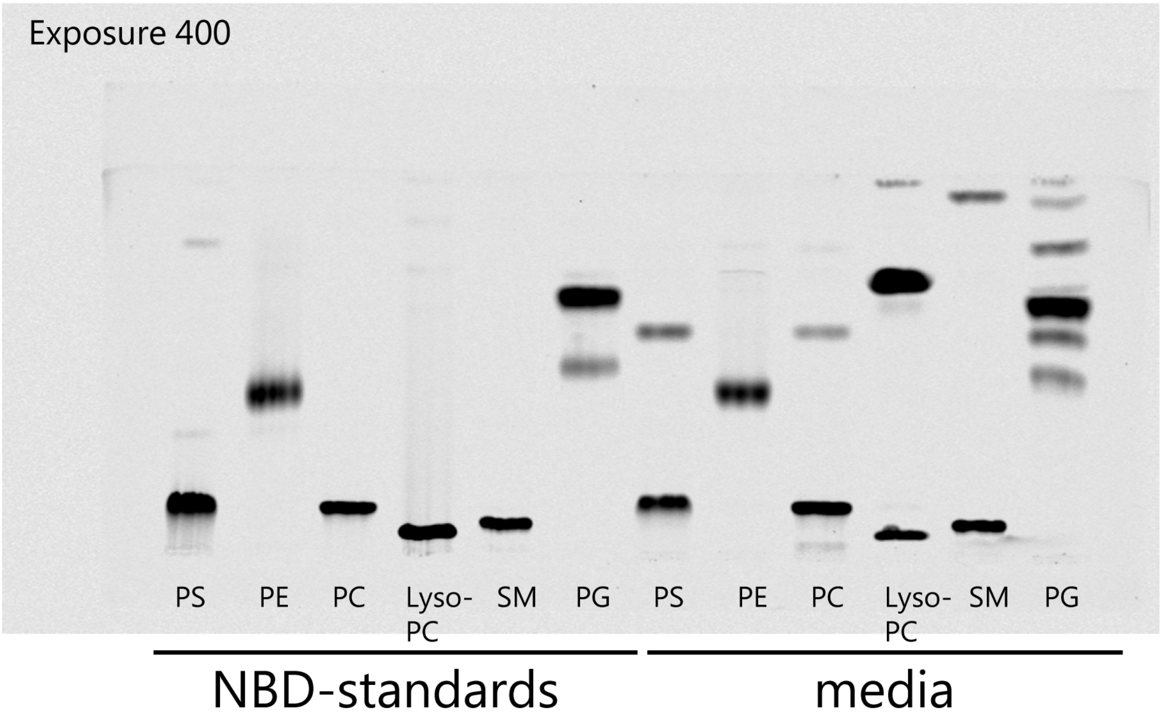

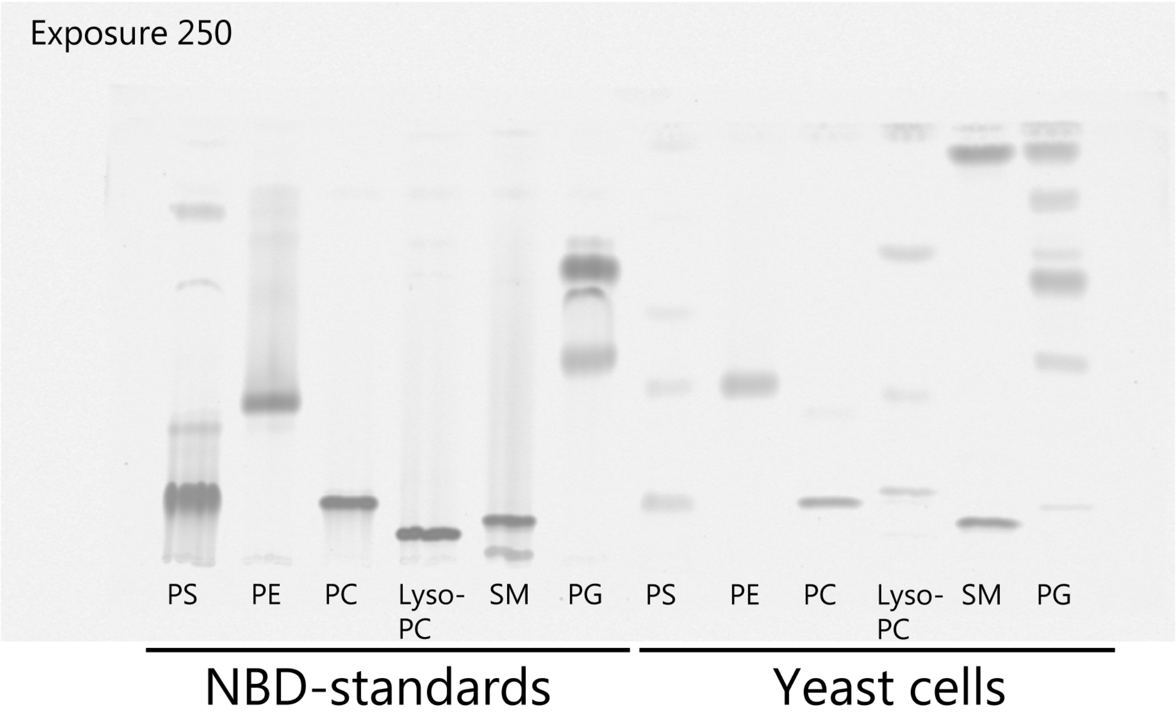
Exposure 250
PS
PE
PC
Lyso-PC
SM
PG
PS
PE
PC
Lyso-PC
SM
PG
NBD-standards
Yeast cells

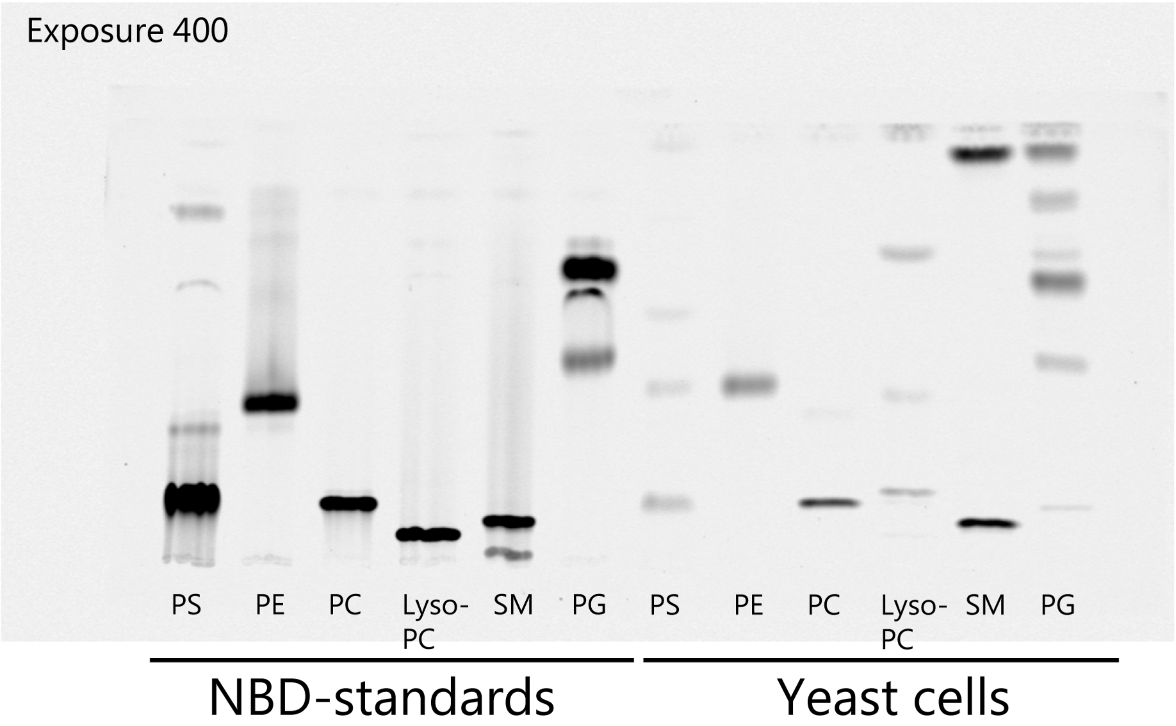
Exposure 400
PS
PE
PC
Lyso-PC
SM
PG
PS
PE
PC
Lyso-PC
SM
PG
NBD-standards
Yeast cells

**Supplementary Dataset S3**: Original scans for the TLC plates used in Supplementary Figure S2 at different exposure times.

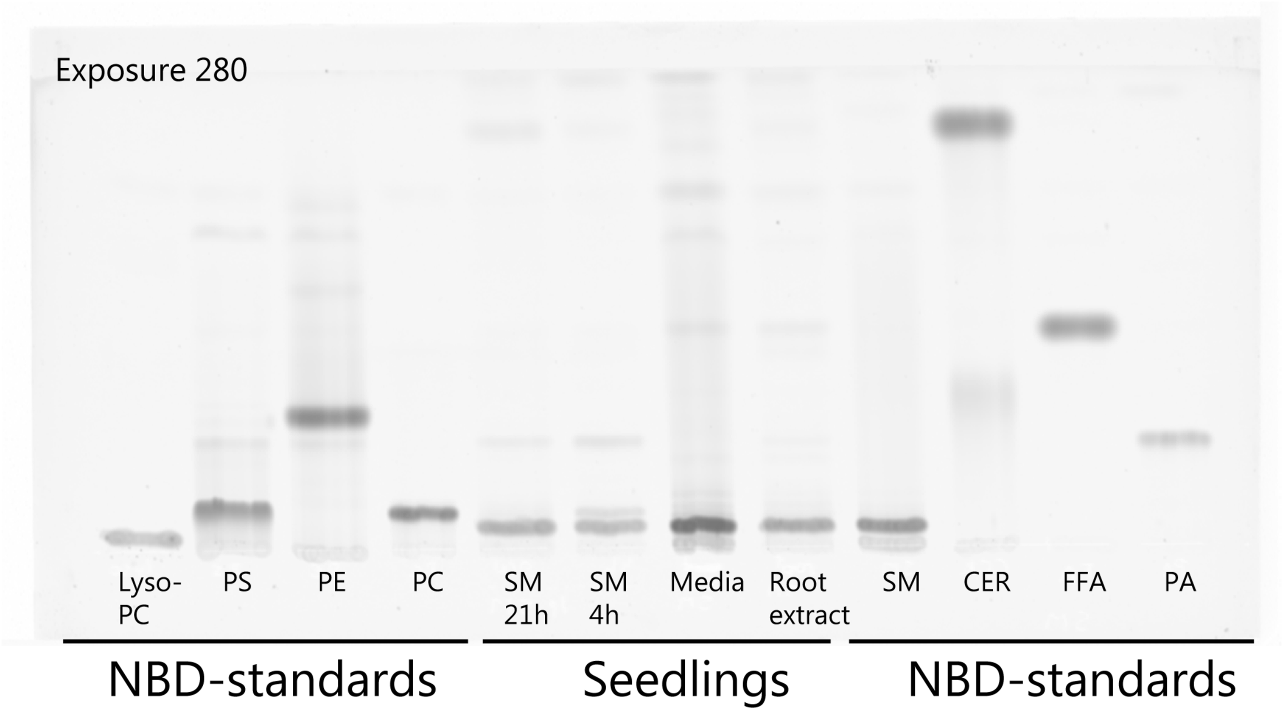


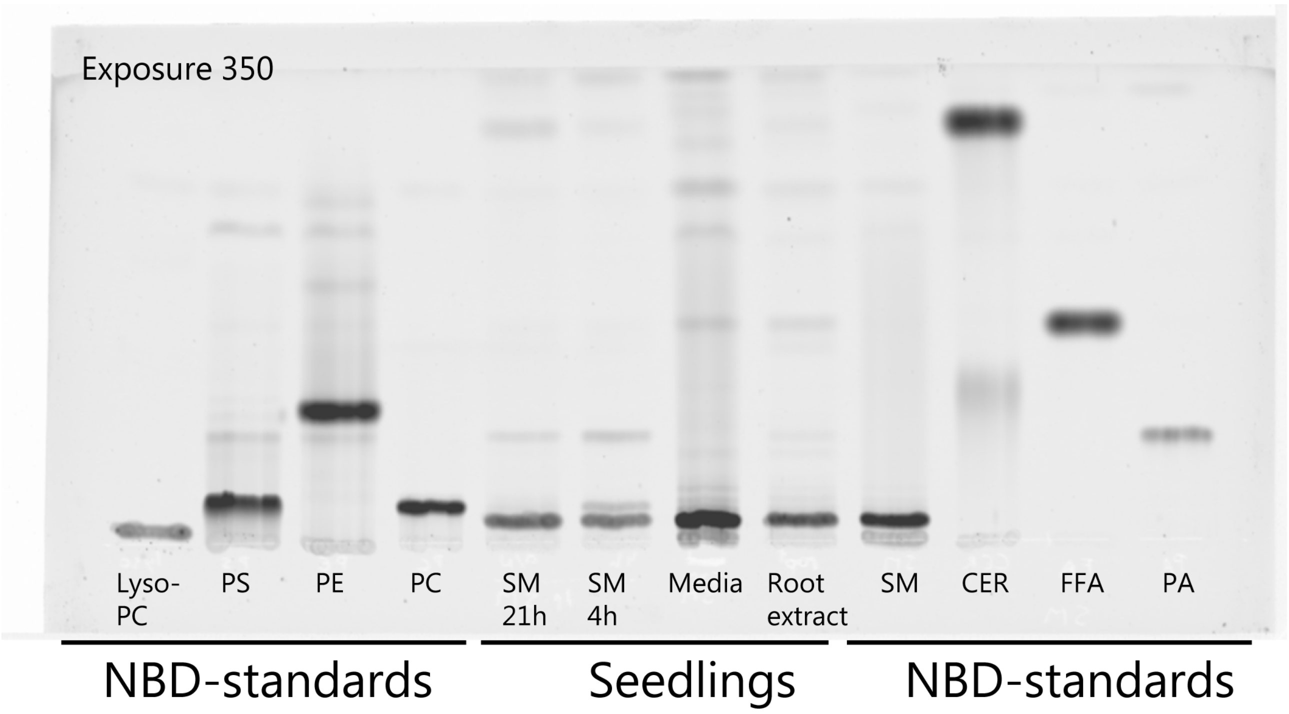

Supplement: Supplementary file 1 — Supplementary Information [file 41598_2017_17742_MOESM1_ESM.pdf]
